# Antibiotic resistance in European wastewater treatment plants mirrors the pattern of clinical antibiotic resistance prevalence

**DOI:** 10.1126/sciadv.aau9124

**Published:** 2019-03-27

**Authors:** Katariina M. M. Pärnänen, Carlos Narciso-da-Rocha, David Kneis, Thomas U. Berendonk, Damiano Cacace, Thi Thuy Do, Christian Elpers, Despo Fatta-Kassinos, Isabel Henriques, Thomas Jaeger, Antti Karkman, Jose Luis Martinez, Stella G. Michael, Irene Michael-Kordatou, Kristin O’Sullivan, Sara Rodriguez-Mozaz, Thomas Schwartz, Hongjie Sheng, Henning Sørum, Robert D. Stedtfeld, James M. Tiedje, Saulo Varela Della Giustina, Fiona Walsh, Ivone Vaz-Moreira, Marko Virta, Célia M. Manaia

**Affiliations:** 1 Department of Microbiology, University of Helsinki, Viikinkaari 9, 00014 University of Helsinki, Finland.; 2 Universidade Católica Portuguesa, CBQF - Centro de Biotecnologia e Química Fina–Laboratório Associado, Escola Superior de Biotecnologia, Rua Arquiteto Lobão Vital, 172, 4200-374 Porto, Portugal.; 3 Technische Universität Dresden, Institute of Hydrobiology, Dresden, Germany.; 4 Department of Biology, Maynooth University, Maynooth, Co. Kildare, Ireland.; 5 Aquantec GmbH, Pfinztalstraße 90, D-76227 Karlsruhe, Germany.; 6 Department of Civil and Environmental Engineering and Nireas–International Water Research Centre, University of Cyprus, P.O. Box 20537, CY-1678 Nicosia, Cyprus.; 7 Department of Biology and CESAM, University of Aveiro, Campus Universitário Santiago, 3810-193 Aveiro, Portugal.; 8 Karlsruhe Institute of Technology (KIT)–Campus North, Institute of Functional Interfaces (IFG), P.O. Box 3640, 76021 Karlsruhe, Germany.; 9 Centro Nacional de Biotecnología, CSIC, Calle Darwin 3, 20049 Madrid, Spain.; 10 Norwegian University of Life Sciences, Faculty of Veterinary Medicine, Department of Food Safety and Infection Biology, Section of Microbiology, Immunology and Parasitology, Post Box 8146 Dep, 0033 Oslo, Norway.; 11 Catalan Institute for Water Research (ICRA), Emili Grahit 101, 17003 Girona, Spain.; 12 Key Laboratory of Soil Environment and Pollution Remediation, Institute of Soil Science, Chinese Academy of Sciences, Nanjing 210008, China.; 13 Department of Civil and Environmental Engineering, Michigan State University, East Lansing, MI 48824, USA.; 14 Center for Microbial Ecology, Department of Plant, Soil and Microbial Sciences, Michigan State University, East Lansing, MI 48824, USA.

## Abstract

Integrated antibiotic resistance (AR) surveillance is one of the objectives of the World Health Organization global action plan on antimicrobial resistance. Urban wastewater treatment plants (UWTPs) are among the most important receptors and sources of environmental AR. On the basis of the consistent observation of an increasing north-to-south clinical AR prevalence in Europe, this study compared the influent and final effluent of 12 UWTPs located in seven countries (Portugal, Spain, Ireland, Cyprus, Germany, Finland, and Norway). Using highly parallel quantitative polymerase chain reaction, we analyzed 229 resistance genes and 25 mobile genetic elements. This first trans-Europe surveillance showed that UWTP AR profiles mirror the AR gradient observed in clinics. Antibiotic use, environmental temperature, and UWTP size were important factors related with resistance persistence and spread in the environment. These results highlight the need to implement regular surveillance and control measures, which may need to be appropriate for the geographic regions.

## Teaser

The antibiotic resistome in European UWTPs mirrors the gradient of north-to-south clinical antibiotic resistance prevalence.

## INTRODUCTION

Antibiotic-resistant bacteria (ARB) can survive the inhibitory action of one or more antibiotics. These ARB reduce the success of infectious disease treatment, which results in important societal and economic costs to human well-being and health (*1*). ARB and their resistance genes are emerging and spreading globally among people, food, animals, plants, and the environment (soil, water, and air) (*2*, *3*). The global action plan on antimicrobial resistance proposed by the World Health Organization (WHO) aims at combating antibiotic resistance at a global scale and across every domain of interface with humans, where the environment is implicitly included (*1*, *4*). Surveillance and the assessment of control measures and the identification of geographical or temporal trends of antibiotic resistance distribution have been consistently shown to be crucial to understanding the impact of antibiotic resistance on human health (*1*, *2*). However, although clinical surveillance efforts are now capable of providing insightful information on antibiotic resistance distribution (*1*, *5*), there is a dearth of data concerning antibiotic resistance in the environment.

Urban wastewater treatment plants (UWTPs) have been recognized as one of the most important routes for propagation of antibiotic resistance from humans to the environment (e.g., fresh water, soil) (*2*). The sewage entering the UWTPs combines the excreta and residues produced in the served area. Therefore, it is expected that the UWTP influents mirror, at least in part, traits of the microbiome of the human population served (*2*, *6*, *7*), including the presence of ARB, resistance genes, and associated mobile genetic elements.

This study aimed at launching the first European antibiotic resistance surveillance in UWTPs, in accordance with objective 2 of the WHO global action plan on antimicrobial resistance: “Strengthen the knowledge and evidence base through surveillance and research” (*4*). The challenges of implementing reliable environmental antibiotic resistance surveillance efforts have been recognized, as the procedures recommended for humans, animals, or food products are not applicable to environmental samples (*2*). Nevertheless, the clinical antibiotic resistance surveillance data stand as an important reference to assess the status of antibiotic resistance in different regions and environmental compartments. Therefore, the current study was designed on the basis of the major trends identified by the European Antimicrobial Resistance Surveillance Network (EARS-Net) currently promoted by the European Centre for Disease Prevention and Control (ECDC). This surveillance integrates data from 30 European countries concerning invasive blood and cerebrospinal fluid isolates of *Escherichia coli*, *Klebsiella pneumoniae*, *Pseudomonas aeruginosa*, *Acinetobacter* spp., *Streptococcus pneumoniae*, *Staphylococcus aureus*, and enterococci (*5*). The 2017 EARS-Net surveillance report (*5*) highlights that, as in previous years, for the period of 2013–2016, “a north-to-south and a west-to-east gradient is evident in Europe,” with a general increase of antibiotic resistance prevalence over this gradient, particularly for the Gram-negative bacteria surveyed. Considering this information, our study included seven countries distributed over the EARS-Net conceptual gradient represented by Portugal, Spain, Cyprus, Ireland, Germany, Norway, and Finland. The relative abundance of different genes was compared in raw influent and final effluent of 12 UWTPs (because of restrictions in sampling authorization, influent samples were not available from Ireland; table S1). Our goal was to obtain an initial overview of the antibiotic resistance status; regular surveillance protocols implemented locally may support robust comparative analyses, where bias associated to sporadic events or regional features can be identified.

**Fig. 1:**
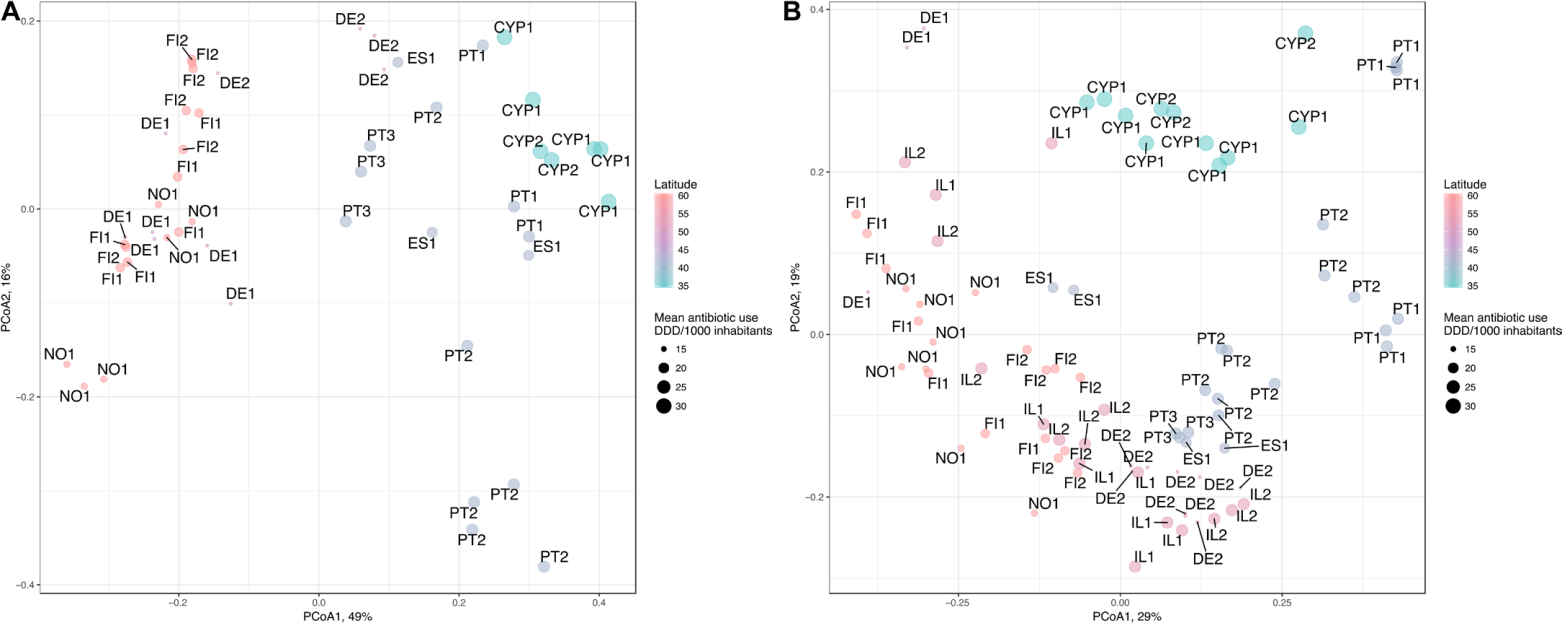
PCoA showing the distribution of resistance and mobile genetic elements using the Bray-Curtis dissimilarity index. (**A**) Influent water from European UWTPs. The H_AC_ and L_AC_ countries clustered separately (*R*^2^ = 0.37, *P* = 0.015). The exception was for three influent samples from German UWTPs that clustered with the H_AC_ samples (DE2 in the figure). (**B**) Effluent water from European UWTPs. The H_AC_ and L_AC_ countries did not cluster together (*R*^2^ = 0.11, *P* = 0.181). The significance of the cluster separation between the H_AC_ and L_AC_ countries was calculated using adonis with 9999 permutations. The points represent individual samples in the ordination, named according to the UWTP, and latitude is indicated by the color gradient. The point size is according to the mean total antibiotic consumption in humans from 2005 to 2015 from ECDC reports consisting of the human consumption of antibacterials for systemic use (ATC group J01) in the community (primary care sector) and the hospital sector expressed as defined daily dose (DDD) per 1000 inhabitants and per day (available at https://ecdc.europa.eu/en/antimicrobial-consumption/database/country-overview) (see also table S3). Countries: H_AC_—PT, Portugal; ES, Spain; CYP, Cyprus; IL, Ireland; L_AC_—DE, Germany; FI, Finland; NO, Norway.

Metagenomics methods, based on shotgun high-throughput sequencing, have been used to analyze the presence of antibiotic resistance genes (ARGs) in ecosystems such as activated sludge, wastewater, and surface water. Whereas these studies are useful to obtain a general view of the most abundant genes, they are not effective for detecting low-abundance genes. As noted before (*6*), abundance does not necessarily correlate with risk, and in the case of antibiotic resistance, surveillance and risk estimation require the implementation of targeted metagenomic techniques (*8*), able to quantify those genes that are of relevance for human health, even when they are present in low amounts that may be nondetectable by using current metagenomics approaches. For these reasons, and also because we sought a quantitative analysis, we used a quantitative polymerase chain reaction (qPCR) array, whose appropriateness as a tool for environmental surveillance has been demonstrated in previous research (*9*, *10*). The array targeted sequences involved in gene transfer and recombination (*n* = 25; integrase, transposase, insertion sequence, and plasmid replicon type) and antibiotic resistance, prevalent in bacterial pathogens (*n* = 229), including multidrug resistance genes (*n* = 39) (table S2). Twenty-four–hour composite samples were collected on each of three consecutive days (Tuesdays, Wednesdays, and Thursdays, in early autumn of 2015, early spring, and early autumn of 2016) at all sampling sites to mitigate biases and improve comparability. In addition, DNA was extracted using a common protocol (table S1), and the highly parallel qPCR analyses were all performed using the same equipment and operational conditions. Of the 384 qPCR array primer pairs, 289, targeting 259 genetic determinants, of which 229 were of resistance genes, 25 of mobile genetic elements, and 5 housekeeping genes, produced amplification in one or more of the analyzed samples, while 95 did not amplify (table S2 and fig. S1). The number of primers for each class of genetic determinants that produced amplification did not differ (*P* > 0.01, Mann-Whitney *U* test) among countries (data not shown). The abundance of antibiotic resistance and mobile genetic elements are, unless otherwise stated, all designated as ARGs. They were normalized to the 16*S* ribosomal RNA (rRNA) gene abundance within the same sample, as described in Materials and Methods. Because this is a measure of the abundance of ARGs per total bacteria, we refer to this ratio as the ARG prevalence. Multidimensional scaling was used to identify the patterns in the distribution of ARGs in the UWTPs’ wastewater (Fig. 1). This analysis indicated that the relative abundance of influent ARGs supported the formation of two distinct groups: one group comprising Portugal, Spain, and Cyprus, and the other group Germany, Norway, and Finland. Although one of the Germany influent samples clustered together with the Portugal, Spain, and Cyprus influent samples, the observed ARG distribution supported the conceptual EARS-Net antibiotic resistance gradient. The relative abundance of ARGs in influents from southern countries (Portugal, Spain, and Cyprus) and northern countries (Germany, Norway, and Finland) clustered separately (*R*^2^ = 0.37, *P* = 0.015) (Fig. 1A). Because the UWTP influent is strongly influenced by human excreta, its resistome can be considered a picture of the general community resistome, hereafter designated “urban resistome.” This resistome can be influenced by, among a myriad of other factors, the general antibiotic resistance prevalence and antibiotic consumption in the community (*2*, *5*). The distribution of urban resistomes based on ARG relative abundance observed in Fig. 1A is consistent with the human antibiotic consumption in those countries in the period 2005–2015 (Fig. 1 and table S3). These two groups of high (H_AC_) and low (L_AC_) antibiotic consumption countries are further compared. Because of the levels of antibiotic use in Ireland (Fig. 1), it was included in the H_AC_ group.

## INSIGHTS INTO THE URBAN RESISTOME

**Fig. 2:**
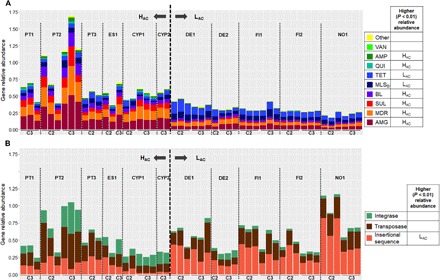
Relative gene abundance observed in influent samples from H_AC_ and L_AC_ countries. Relative abundance of (**A**) resistance genes and (**B**) mobile genetic elements. The data refer to the sum of relative abundance of amplification (ratio ARG or MGE copy number: 16*S* rRNA gene copy) for a given pair of primers, organized in classes of “resistance” or “transfer and recombination.” In the legend, for each gene class, the country group, H_AC_ or L_AC_, with significantly higher relative abundance (*P* < 0.01, Mann-Whitney *U* test) is indicated. Samples are organized according to the sampling campaign (C2, spring 2016; C3, autumn 2016), divided by H_AC_ and L_AC_. Resistance categories: AMG (aminoglycosides), MDR (multidrug resistance), SUL (sulfonamides), BL (β-lactams), MLS_B_, TET (tetracycline), QUI (quinolones), AMP (amphenicols), VAN (vancomycin), and others. Note: Ireland data are missing because of restrictions on influent wastewater sample collection.

Most of the ARGs detected in the influents corresponded to genes with widespread environmental distribution encoding resistance against first-generation antibiotics. All influent samples contained genes conferring resistance to aminoglycosides (*aadA* and *strB*), β-lactams (*bla_GES_*, *bla_OXA_*, and *bla_VEB_*), macrolide–lincosamide–streptogramin B (MLS_B_) (*ereA*, *ermF*, and *matA*/*mel*), sulfonamides (*sul1*), tetracyclines (*tetM* and *tetQ*), and multidrug resistance (*qacEdelta1* and *qacH*). Moreover, the signatures of various genetic elements involved in gene transfer and recombination (*intI1*, *tnpA*, *Tp614*, *ISAba3*, *ISPps*, and *ISSm2*) were present in all influent samples (table S2). Regarding ARGs of high concern in clinical settings (*bla*_*NDM*-*1*_, *bla_KPC_*, *bla_VIM_*, *bla_IMP_*, *mcr-1*, *mecA*, or *vanA*), *bla_IMP_* and *vanA* were sporadically detected in the influent of different countries. The *bla_VIM_* gene was detected in Portugal, Spain, Cyprus, and Germany influents, and *bla_KPC_* in Portugal and Spain influents and sporadically in effluent samples. The ARG distribution that separated the H_AC_ from the L_AC_ countries was explained by a significantly (*P* < 0.01, Mann-Whitney *U* test) higher relative abundance of most resistance classes in H_AC_ countries (Fig. 2A). Specifically, the relative abundance of gene families conferring resistance to aminoglycosides, sulfonamides, β-lactams, quinolones, amphenicols, and multidrug resistance was higher in the H_AC_ countries (Portugal, Spain, Cyprus, and Ireland) than in the L_AC_ countries (*P* < 0.01, Mann-Whitney *U* test). The opposite was observed for tetracycline and MLS_B_ resistance genes (Fig. 2A). The difference in the relative abundance of determinants related to gene transfer and recombination was also noticeable. Specifically, the relative abundance of insertion sequences was higher in the L_AC_ than in the H_AC_ countries (*P* < 0.01, Mann-Whitney *U* test) (Fig. 2B). The relative abundance of genes encoding resistance to amphenicols, β-lactams, sulfonamides, and integrase enzymes presented significantly higher daily variations over the 3 days of the same sampling campaign in the H_AC_ than in the L_AC_ countries (*P* < 0.01, Mann-Whitney *U* test). This effect might have been due to the comparatively smaller size of the UWTPs of the H_AC_ group in comparison with those of the L_AC_ group (≤200 versus ≥300 population-equivalent and <45 versus >100 m^3^/day) (table S4). In smaller UWTPs, peaks of exogenous ARGs may have a more pronounced effect. Given the contrasts observed between both groups of countries, we were interested in investigating whether wastewater treatment attenuates those contrasts, resulting in similar ARG abundances in all the UWTP effluents.

## INSIGHTS INTO THE FINAL EFFLUENT RESISTOME

**Fig. 3:**
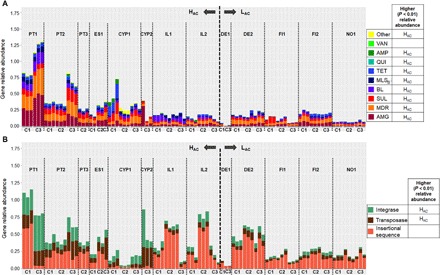
Relative gene abundance observed in effluent samples from H_AC_ and L_AC_ countries. Relative abundance of (**A**) resistance genes and (**B**) mobile genetic elements. The data refer to the sum of relative abundance of amplification (ratio ARG or MGE copy number: 16*S* rRNA gene copy) for a given pair of primers organized in classes of resistance or transfer and recombination. In the legend, for each gene class, the country group, H_AC_ or L_AC_, with significantly higher relative abundance (*P* < 0.01, Mann-Whitney *U* test) is indicated. Samples are organized according to the sampling campaign (C1, autumn 2015; C2, spring 2016; C3, autumn 2016), divided by H_AC_ and L_AC_. Resistance categories: AMG (aminoglycosides), MDR (multidrug resistance), SUL (sulfonamides), BL (β-lactams), MLS_B_, TET (tetracycline), QUI (quinolones), AMP (amphenicols), VAN (vancomycin), and others.

**Fig. 4:**
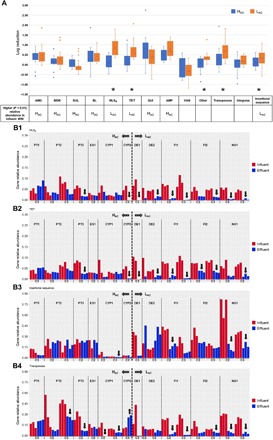
Variation in average ARG prevalence between influent and effluent [log_10_(influent − effluent)] in H_AC_ and L_AC_ countries. (**A**) Comparison of ARG log reduction values in UWTPs of the H_AC_ and L_AC_ countries, where zero indicates that treatment did not affect the cumulative ARG relative abundance, positive values indicate a reduction, and negative values indicate an increase. The classes for which H_AC_ or L_AC_ presented higher relative abundance in the influent (*P* < 0.01, Mann-Whitney *U* test) are indicated in the table at the bottom. Asterisks indicate statistically significant differences between H_AC_ and L_AC_ log reduction values, and these cases are detailed in (B). (**B**) Comparison of average ARG relative abundance in influent and final effluent, for the classes with significantly different log reduction values in the H_AC_ and L_AC_ samples: (B1) MLS_B_, (B2) tetracyclines, (B3) insertion sequences, and (B4) transposase. The arrows indicate significant (*P* < 0.01; Welch’s *t* test) increases (↑) or decreases (↓) after treatment (C1, autumn of 2015; C2, spring of 2016; C3, autumn of 2016). WW, wastewater.

The comparative analyses of the final effluents aimed at assessing whether, irrespective of the input, the wastewater treatment could reduce the levels of ARGs to a similar baseline level across the different UWTPs examined. Similar to the influent resistomes, contrasts were observed between the H_AC_ and L_AC_ effluent samples. For most resistance classes (aminoglycoside, sulfonamides, β-lactams, MLS_B_, tetracycline, quinolones, amphenicols, and multidrug resistance), the H_AC_ final effluents presented significantly higher relative abundance values than the L_AC_ final effluents (*P* < 0.01, Mann-Whitney *U* test) (Fig. 3A). This was also observed for genes encoding integrases and transposases (Fig. 3B). The relative abundance of tetracycline and MLS_B_ resistance genes and insertion sequences that was higher in the L_AC_ than in the H_AC_ influents (Fig. 2) became lower in the final effluents. The relative abundance of tetracycline and MLS_B_ resistance genes changed after treatment and was significantly higher in the H_AC_ than in the L_AC_ countries (*P* < 0.01, Mann-Whitney *U* test) (Fig. 3). These shifts were in agreement with the differences observed in the prevalence decrease of different ARG classes in the L_AC_ and H_AC_ countries (Fig. 4A). The classes with sharper decreases of prevalence values in the L_AC_ compared to the H_AC_ countries were the ARGs encoding resistance against tetracyclines and MLS_B_ and the insertion sequences and transposase encoding genes (*P* < 0.01, Mann-Whitney *U* test). Noticeably, most of these classes of genes had a higher prevalence in the influent in the L_AC_ than in the H_AC_ samples (Figs. 2 and 4). Also, in the final effluent data, the richness of the MLS_B_ genes was significantly different (*P* < 0.01, Mann-Whitney *U* test) in Portugal compared with Ireland and the L_AC_ countries (data not shown). Although the relative abundance of most ARGs decreased after treatment in both the H_AC_ and L_AC_ countries, effluents from the H_AC_ group had a significantly higher relative abundance of most of the ARG classes, with the exception of vancomycin resistance, integrase, and transposase encoding genes (Fig. 4). Noticeably, the exclusion of Cyprus samples, apparent outsiders in the principal coordinates analysis (PCoA) (Fig. 1), from the analyses did not change this scenario, except for vancomycin resistance (data not shown). The prevalence values varied differently for distinct ARG classes. While for the H_AC_ the prevalence of aminoglycosides, amphenicols, and quinolones resistance classes had higher reduction than the other classes, for the L_AC_ the prevalence of amphenicols, MLS_B_, tetracyclines, and the mobile genetic elements were the most reduced (data not shown). In all countries, vancomycin resistance genes were observed to be slightly enriched after treatment, and they sometimes shifted from levels below the detection limit to quantifiable amounts. Also, sulfonamide resistance genes were observed to be slightly enriched in the L_AC_ countries (Fig. 4A).

Core wastewater ARGs, meaning those present in all wastewater samples analyzed, from both influent and effluent, comprised *qacEdelta1* and *sul1* resistance genes and the *ISSm2* insertion sequence (table S2). In addition, the ARGs related with resistance to aminoglycosides (*aadA* and *strB*), β-lactams (*bla_OXA_*), MLS_B_ (*ermF*), sulfonamides (*sul2*), tetracycline (*tetW*), multidrug resistance (*qacH*), and mobile genetic elements, such as transposase (*tnpA*), integrase (*intI1*), and insertion sequences (*ISAba3* and *ISPps*), persisted after treatment in >90% of the analyzed samples (table S2). Resistance genes considered of highest concern in clinical settings analyzed in this study were not detected in the final effluents examined. Sporadic exceptions were, e.g., *mecA* detected in final effluent samples from Cyprus or *bla_IMP_* and *vanA* detected in both influent and final effluent samples in all countries (except *bla_IMP_* in Cyprus).

## INFERENCE ABOUT POSSIBLE ARGS INDICATORS IN WASTEWATER

Surveillance can be performed with two aims, both requiring different approaches and targeting distinct ARGs. One refers to the evaluation of the overall ARGs burden, a measure of the degree of contaminant ARGs occurring in a given environment. This surveillance relies on the measurement of the total resistome and its quantitative variations and pattern fluctuations. The other, which specifically focuses on biological hazards, relies on the targeted analyses of ARGs of special clinical concern or emerging in the environment. These are normally at lower abundance than most environmental ARGs, and their detection may require the use of approaches that increase sensitivity to ensure detection at very low levels (*11*). Despite the importance of wastewater surveillance to assess geographic trends or, at long-term, to measure the impacts of control measures, the monitoring of a high number of ARGs may be limiting due to the cost and training required for analysis on a routine basis. The use of the traditional fecal bacteria enumeration to assess the microbiological water quality may be of limited value for resistance monitoring. We could not find significant correlations between the antibiotic-resistant counts of culturable bacteria (table S4), performed with these same samples, and the ARG quantification. High-throughput methods have the potential to generate information from which indicators can be inferred. The data gathered in this study, which cover a large number of ARGs and geographic regions, were scanned for suitable predictors of the total ARG abundance in UWTP effluents (Table 1). The suggested indicator primers either amplified genes encoding resistance to first-generation antibiotics or associated to mobile genetic elements, with high prevalence in wastewater (Table 1). Among these were *aadA*, *qacEdelta1*, *ermF*, and *intI1*. Noticeably, some of these elements are associated with class 1 integrons, recognized as markers of anthropogenic impact (*12*). On the basis of these data, a mini-array could be designed, aimed at implementing operational screening that covers major target drug classes (amphenicols, β-lactam, MLS_B_, sulfonamide, and tetracycline) or functional groups (insertion sequence and transposase). Notably, the total abundance of resistance genes could be predicted using a subset of primers. For example, the data from assay AY159 (*qacEdelta1*) alone explain almost 90% of the variance in total ARG abundance. Using a linear model with three inputs, e.g., AY167 (*aadA*), AY23 (*ermF*), and AY337 (*intI1*), the predictive power could be moderately increased (adjusted *R*^2^ = 0.95). This information provides a promising direction for the future wastewater surveillance with a substantial workload and cost reduction as well as ease of data analyses when compared with nontargeted and high-throughput approaches.

**Table 1:** Candidate indicator assays for major gene classes in UWTP effluents. Assays that yielded the highest amplification in the largest number of samples are reported on the left. Assays with the most representative outcome in terms of correlation with the per-class mean are reported on the right (rho: Spearman’s rank correlation coefficient). Primer pairs corresponding to the assay are listed in table S2.

**Gene class** **(no. of assays)**	**Genes dominating the class**			**Most representative genes**		
	**Gene**	**Assay ID**	**% of samples**	**Gene**	**Assay ID**	**Rho**
Aminoglycoside (24)	*aadA*	AY167	62.9			0.96
Amphenicol (14)	*cmxA*	AY129	27.1	*cmlA*	AY127	0.74
β-Lactam (61)	*blaOXA*	AY44	34.5			0.76
MDR (40)	*qacEdelta1*	AY159	92.1			0.97
MLS_B_ (30)	*ermF*	AY23	68.6			0.81
Quinolone (3)	*qnrSrtF11*	AY6	100.0			1.00
Sulfonamide (9)	*sul1*	AY363	73.0			0.82
Tetracycline (34)	*tetQ*	AY185	59.6	*tetX*	AY196	0.73
Insertion sequence (9)	*ISPps*	AY369	86.5			0.98
Integrase (7)	*intI1*	AY336	80.9			0.96
Transposase (10)	*tnpA*	AY202	79.3			0.86

## DISCUSSION

A major expectation of this first integrated surveillance of European wastewaters was to assess whether the relative abundance of urban wastewater resistome would follow the same trend observed in the prevalence of antibiotic resistance in clinical isolates, also coincident with antibiotic use profiles herein designated by H_AC_ and L_AC_. The distribution of ARGs in the urban resistome showed the separation of the north and south countries studied, which is consistent with what has been shown for clinical isolates (*5*). The results suggest that studied UWTPs, based on activated sludge secondary treatment processes, with different configurations and, in a few cases, with a tertiary treatment or simply a disinfection stage, were able to reduce the relative abundance of all examined ARG classes, except genes encoding resistance to vancomycin (Fig. 4). However, the ARG burden after wastewater treatment was significantly higher in the south (H_AC_) than in the north (L_AC_) countries (Fig. 3). As a consequence, it is hypothesized that the final effluents of the H_AC_ countries may have a higher impact on the receiving environment than those of the L_AC_ countries. A result that deserves future research is the basis why the ARG prevalence decrease is higher in some cases in the L_AC_ countries. This observation may be related to the dynamics of the ARG bacterial hosts during treatment (*13*, *14*), influenced by operational conditions (e.g., temperature) and microbiota composition. In addition, or alternatively, it may be a consequence of the differences in the wastewater treatment reactors, which are larger in the L_AC_ countries than in the H_AC_ countries (table S4). Although the diversity of ARGs in activated sludge samples, assessed based on metagenomics, seems to be fairly conserved in different wastewater treatment plants (*15*, *16*), investigation of the enriched microbiota and operational conditions on ARG reduction seems warranted. The potential influence of the presence of selective pressures, particularly antibiotic residues, during treatment has been suggested in different studies as capable of influencing the fate of antibiotic resistance (*17*–*20*). The analyses of more than 50 antibiotic residues in the same effluent samples used in this study did not show any correlation with relative abundance of ARGs [(Rodriguez-Mozaz *et al*., in preparation). Similarly, no statistically significant correlations could be established between the relative abundance of ARGs in the final effluents and country-level information on antibiotic consumption in the primary care sector [data from ESAC-Net; (*21*)]. The influence of the treatment process or of specific operational conditions on ARG relative abundance was beyond the scope of this surveillance. However, the variations in ARG prevalence observed in the H_AC_ and L_AC_ countries revealed by this study are highly relevant and novel findings that deserve future research investigation.

The evolution and spread of antibiotic resistance is a complex process, resulting from the interplay of different and often confounding variables. The H_AC_ and L_AC_ countries differed in the rate of antibiotic use in humans, but also in other, probably non-negligible factors, such as temperature, precipitation, or antibiotic use in pets and in livestock. Although the antibiotics used in livestock production are not discharged into the municipal sewage system, these residues may contribute to an overall increase of antibiotic or antibiotic resistance load in that region. The H_AC_ countries, excluding Ireland, also had higher animal antibiotic use and higher average minimal temperatures (biomass, 187.2 to 425.8 mg/kg; >11°C) than the L_AC_ countries (biomass, 3.7 to 179.7 mg/kg; <5°C) (fig. S2). Curiously, Ireland, with lower temperature and animal antibiotic use, had final effluents with lower relative ARG abundance (*P* < 0.01, Mann-Whitney *U* test) than the other H_AC_ countries, except Spain. Germany, on the contrary, had higher antibiotic animal use than the other L_AC_ countries; however, its relative abundance of ARGs was not significantly different from other L_AC_ countries (*P* < 0.01, Mann-Whitney *U* test).

**Fig. 5:**
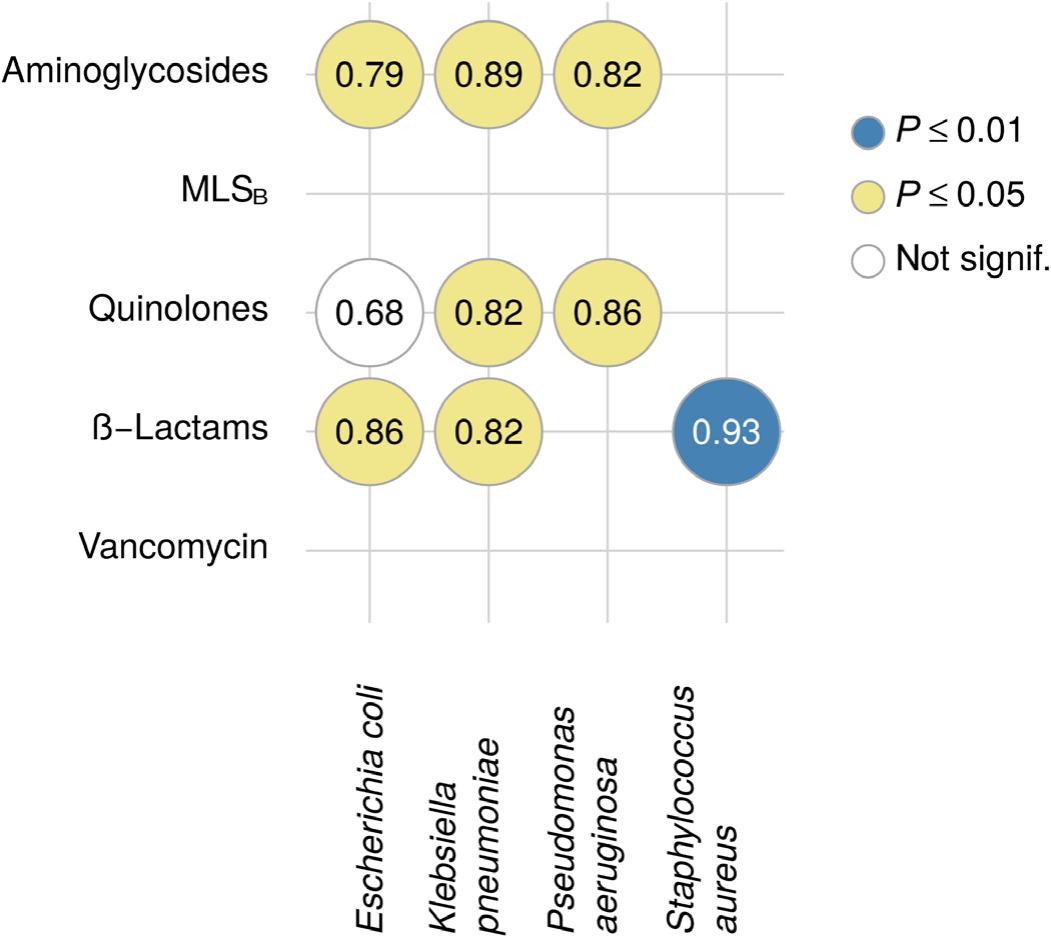
Association between the relative abundance of ARGs in UWTP effluents (aggregated by drug classes) and country-level data on phenotypic resistance of clinical isolates [data from EARS-Net,]. (*31*) Numbers represent Spearman’s rank correlations; color codes indicate statistical significance. No statistically significant correlations were observed for *Acinetobacter* spp., *Enterococcus faecium*, or *S. pneumoniae*.

Although the north-to-south clinical antibiotic resistance gradient reported by the EARS-Net was also present in the European wastewaters analyzed in this study, we were interested in assessing the possible correlation between the clinical settings and the final effluents of the UWTPs studied. The relative abundance of several ARGs was significantly correlated with the prevalence of phenotypic resistance in clinical isolates of *E. coli*, *K. pneumoniae*, *P. aeruginosa*, or *S. aureus* (Fig. 5). This result is interesting and supported by the recognized ubiquity of these bacterial groups. Ubiquity and environmental fitness may be key concepts when discussing antibiotic resistance in the environment. Among the bacterial groups surveyed by EARS-Net, those that show an evident north-to-south gradient of resistance prevalence are ubiquitous bacteria that can thrive in the human body as well as in wastewater. These bacteria have optimal growth temperatures above 30°C (*E. coli*, 37°C; *K. pneumoniae*, 30° to 35°C; *P. aeruginosa*, 37°C; and *Acinetobacter* spp., 33° to 35°C) (*22*). Hence, it is not surprising that these bacteria survive better in the environment in regions with warmer temperatures. In a recent study, MacFadden *et al*. (*23*) demonstrated that an increase of 10°C across regions coincided with higher antibiotic resistance percentage of 4.2% for *E. coli*, 2.2% for *K. pneumoniae*, and 2.7% for *S. aureus*. Together with antibiotic use, and eventually with higher impact than that of antibiotic use, temperature may be a major driver of antibiotic resistance persistence and proliferation in the environment. Curiously, the three UWTP Germany samples that clustered with H_AC_ influent samples (DE2 in Fig. 1) were from the treatment plant located in a region with the highest average temperature, which also presented higher culturable bacterial counts than the L_AC_ countries (table S4).

This is the first European integrated antibiotic resistance surveillance in wastewaters. Although this survey represents a limited number of UWTPs at the European scale, the fact that they were selected on the basis of the single criterion of location along the north-to-south gradient is supportive of our conclusions. The results not only reinforce the strong relationship between clinical and environmental antibiotic resistance but also signal the importance of taking societal and climate factors (e.g., temperature or precipitation) in design of possible strategies to control antibiotic resistance. The importance of surveillance to improve antibiotic resistance control is also evident in this study. However, we have concluded that the use of traditional fecal indicators may have a limited capacity to provide reliable comparisons of antibiotic resistance status in wastewaters. Although culture-dependent methods continue to have their place in water quality monitoring, they may lack the necessary sensitivity for comparative purposes. Therefore, another interesting outcome of this comprehensive study is the possibility of designing simplified and low-cost surveillance protocols. These protocols, supported by high-throughput data, can be feasibly used on a routine basis for wastewater antibiotic resistance monitoring worldwide.

## MATERIALS AND METHODS

### Sampling and sample processing

The study included seven European countries, where UWTP samples were collected over three sampling campaigns in early autumn 2015 and 2016 and early spring 2016. Volumes between 1 and 2 liters were collected, in sterile flasks, and stored at 4ºC until analysis for a maximum of 12 hours. In each sampling campaign, were collected three 24-hour composite samples, one per day, over three consecutive days (Tuesday, Wednesday, and Thursday) (table S1). Geographic, demographic, and technical characterization provided by the UWTPs is available in table S4. All participants followed a common protocol for sampling, sample processing, and DNA extraction.

### DNA extraction and processing

All wastewater samples (*n* = 168) were filtered in triplicate through sterile polycarbonate membranes (0.22-μm porosity; Whatman, UK) and stored at −80ºC until DNA extraction. Volumes of 25 to 50 ml of influent wastewater samples or of 150 to 400 ml of final effluent wastewater samples were filtered. Total DNA was extracted in triplicate, at once for the same sampling campaign, using the PowerWater DNA Isolation Kit (MO BIO Laboratories Inc., CA, USA) according to the manufacturer’s instructions, and quantified using the Qubit 3.0 Fluorometer (Thermo Fisher Scientific, USA). For each sampling date, a DNA pool from the triplicates was prepared, resulting in one DNA extract per day and UWTP and three extracts per sampling campaign. DNA extract pools were shipped to the Michigan State University and run using the qPCR array, as previously described (*24*). Each partner kept an aliquot of the DNA pools for additional analyses as needed.

### qPCR array

The qPCR array contained 384 primer sets as previously described (*10*), including some additional primer sets: 16*S* rRNA gene (AY2), *bla*_*CTX*-M_ (AY326 and AY360), *bla_OXA_* (AY361), *bla_SHV_* (AY272), *bla_TEM_* (AY3), *bla_VIM_* (AY260), *mecA* (AY284), *qnrSrtF11* (AY6), *sul1* (AY110), *tetM* (AY357), *vanA* (AY4; AY368), *mcr-1* (AY80), and *intI1* (AY45) (table S2). The concentration and quality of the DNA samples were verified using the Qubit 3.0 Fluorometer (Thermo Fisher Scientific, USA). Only the samples fulfilling the quality criteria were processed. The qPCR array uses a microfluidic SmartChip Multisample Nanodispenser (WaferGen at the time of the study, now Takara) to load primer sets and samples from 384-well plates into a SmartChip (Takara) with 5 184-reaction wells. The same 384-well plate of primers was used for all experiments, and three technical replicates were run for each sample. Samples were diluted to have the same mass of DNA per reaction. Following amplification on the SmartChip Real-Time PCR cycler, *C*_t_ values were calculated using default parameters provided with the SmartChip analysis software.

### Cultivable bacteria

Wastewater samples were characterized for the fecal coliform and enterococci counts, two groups of bacteria frequently used as microbiological indicators of water quality. The bacterial enumeration was performed on membrane fecal coliform agar (mFC) or on m-Enterococcus agar and on these culture media supplemented with one of the following antibiotics: amoxicillin (32 mg/liter), tetracycline (TET; 16 mg/liter), or ciprofloxacin (1 mg/liter). A volume of 1 ml of wastewater or of the adequate serial dilution was filtered through cellulose nitrate membranes (0.22-μm porosity; Sartorius Stedim Biotech, Göttingen, Germany), placed onto the adequate culture medium and incubated at 30ºC for 24 hours for fecal coliforms or 48 hours for enterococci. Counts were done in triplicate, and the results were used as part of wastewater characterization provided in table S4.

### Data analysis

The quality criteria used to select the qPCR array data to be used in the analyses were (i) samples with at least two replicates, (ii) samples with more than 5 ng of DNA, and (iii) quantifications with a *C*_t_ ≤ 27. A *C*_t_ value of 27 corresponded to a limit of quantification lower than 100 gene copies (*25*). The qPCR array semiquantitative analyses did not support estimations based on gene abundance per volume of water because calibration curves were not obtained for each gene. However, for a subset of 10 genes, calibration curves that supported the assessment of the relationship between relative (16*S* rRNA based) and absolute quantification (fig. S3) were obtained. Some samples (Portugal) used for the array were also tested by real-time qPCR for the quantification of selected genes (fig. S4). Those analyses allowed us to conclude that the overall trends are similar between the quantitative qPCR and the semiquantitative qPCR array.

The 384 primer sets target 259 different genetic determinants. These comprised 229 ARGs organized in classes according to the class of antibiotics to which they confer resistance (AMG, aminoglycosides; SUL, sulfonamides; BL, β-lactams; MLS_B_; TET, tetracycline; QUI, quinolones; AMP, amphenicols; VAN, vancomycin; and others), as MDR (multidrug resistance) when conferring resistance to more than one class of antibiotics; 16 genetic transfer and recombination elements (integrase, transposase, and insertion sequence); 9 plasmid-associated genetic determinants; and 5 housekeeping genes. The plasmid-associated assays were excluded from the analyses because of the poor coverage (table S2). All the analyses were performed considering the remaining 375 primer pairs independently. The results of amplicons’ relative abundance were calculated using the *C*_t_ values of the reference gene and the genes of interest, applying the following formula: (2^(*C*t_reference gene_ − *C*t_target gene_)^) (*26*).

The statistical analyses were performed using the R environment v3.4.0. To compare the average values between two groups, Mann-Whitney *U* tests (*27*) were performed. Correlations between the relative abundance of each gene and the sum of the relative abundance of all genes from the same group (Table 1) were computed using Spearman’s rank-based approach (*28*). Statistical modeling of the total abundance of ARGs involved a power transformation of the gene’s relative abundances and the complete removal of zero-inflated assays (no measurable amplification in >25% of the samples).

All reported *P* values were adjusted for multiple testing according to the respective hypotheses (*29*). To calculate the log reduction of the abundance of resistance and genetic transfer and recombination classes, the formula log_10_(influent relative abundance) − log_10_(effluent relative abundance) was applied, and Mann-Whitney *U* test was used to find significant differences between H_AC_ and L_AC_. PCoA was calculated using the Bray-Curtis dissimilarities calculated using the package vegan v2.4 (*30*) and the cmdscale command from stats package. The latitude was defined by the latitude of the city of the UWTP. The data for human consumption were collected from the antimicrobial consumption database (available at https://ecdc.europa.eu/en/antimicrobial-consumption/database/country-overview), considering the consumption of antibacterials for systemic use (ATC group J01) in the community (primary care sector) and the hospital sector expressed as defined daily dose (DDD) per 1000 inhabitants and per day using the mean from years 2005–2015.

To study the association between relative ARG abundance in UWTP effluents and phenotypic resistance in the primary care sector [data from EARS-Net, (*31*); Fig. 5], the information from the two sources was matched in terms of target antibiotics. Specifically, phenotypic resistance against penicillins, aminopenicillins, third-generation cephalosporins, and methicillin was classified as (and compared to) genotypic β-lactam resistance. Phenotypic macrolide resistance was compared with the relative abundance of ARGs conferring resistance to MLS_B_.

## Supplementary Material

http://advances.sciencemag.org/cgi/content/full/5/3/eaau9124/DC1

Download PDF

Data file S1

Data file S2

Data file S3

## SUPPLEMENTARY MATERIALS

Supplementary material for this article is available at http://advances.sciencemag.org/cgi/content/full/5/3/eaau9124/DC1 Fig. S1. Average richness values (number of positive assays) for the different influent and effluent wastewater samples from high (H_AC_) and low (L_AC_) antibiotic consumption countries, for resistance genes and mobile genetic elements. Fig. S2. Food-producing animals’ antibiotics consumption [expressed in biomass (mg/kg), values for 2013] and average maximal and minimal annual temperature and precipitation (yellow, average *T*_min_ >6°C; blue, average *T*_max_ <5°C). Fig. S3. Average abundance (copies/ml; upper bars; left-hand legend) and prevalence values (gene copies/16*S* rRNA gene copies; lower bars; right-hand legend) for the different influent (Inf) and effluent (Eff) wastewater samples from high (H_AC_) and low (L_AC_) antibiotic consumption countries, determined on the basis of qPCR array for the genes: 16*S* rRNA, *intI1*, *bla_OXA_*, *sul1*, *tetM,* *ermF,* *aadA,* *tnpA,* and *qacEdelta1*. Fig. S4. Average abundance (copies/ml; upper bars; left-hand legend) and prevalence values (gene copies/16*S* rRNA copies; lower bars; right-hand legend) calculated by traditional real-time qPCR and qPCR array for Portuguese influent and effluent wastewater samples. Table S1. Influent and effluent wastewater samples used in the study. Table S2. qPCR primer sets and the percentage of samples that gave positive results for the influent and effluent wastewater samples. Table S3. Consumption of antibacterials for systemic use (ATC group J01) in the community (primary care sector) in different European countries from 2005 to 2015 (see also Fig. 1), defined as daily dose per 1000 inhabitants and per day. Table S4. Characterization of the UWTPs examined in this study, in terms of dimension, geographic conditions, treatment, and microbiological indicators. Data S1. qPCR array data. Data S2. List of samples. Data S3. List of assays. Reference (*32*)
