# Supplementary material for: Antibiotic resistance in European wastewater treatment plants mirrors the pattern of clinical antibiotic resistance prevalence
Source: Sci Adv. 2019 Mar 27;5(3):eaau9124. doi: 10.1126/sciadv.aau9124 (PMC6436925; doi:10.1126/sciadv.aau9124)
Supplement: http://advances.sciencemag.org/cgi/content/full/5/3/eaau9124/DC1 [file supp_5_3_eaau9124__index.html]

Science Advances | Science Advances

## Supplementary Materials

**The PDF file includes:**

- Fig. S1. Average richness values (number of positive assays) for the different influent and effluent wastewater samples from high (HAC) and low (LAC) antibiotic consumption countries, for resistance genes and mobile genetic elements.
- Fig. S2. Food-producing animals’ antibiotics consumption expressed in biomass (mg/kg), values for 2013 and average maximal and minimal annual temperature and precipitation (yellow, average *T*min >6°C; blue, average *T*max <5°C).
- Fig. S3. Average abundance (copies/ml; upper bars; left-hand legend) and prevalence values (gene copies/16*S* rRNA gene copies; lower bars; right-hand legend) for the different influent (Inf) and effluent (Eff) wastewater samples from high (HAC) and low (LAC) antibiotic consumption countries, determined on the basis of qPCR array for the genes: 16*S* rRNA, *intI1*, *blaOXA*, *sul1*, *tetM*, *ermF*, *aadA*, *tnpA*, and *qacEdelta1*.
- Fig. S4. Average abundance (copies/ml; upper bars; left-hand legend) and prevalence values (gene copies/16*S* rRNA copies; lower bars; right-hand legend) calculated by traditional real-time qPCR and qPCR array for Portuguese influent and effluent wastewater samples.
- Table S1. Influent and effluent wastewater samples used in the study.
- Table S2. qPCR primer sets and the percentage of samples that gave positive results for the influent and effluent wastewater samples.
- Table S3. Consumption of antibacterials for systemic use (ATC group J01) in the community (primary care sector) in different European countries from 2005 to 2015 (see also Fig. 1), defined as daily dose per 1000 inhabitants and per day.
- Table S4. Characterization of the UWTPs examined in this study, in terms of dimension, geographic conditions, treatment, and microbiological indicators.
- Reference (*32*)

Download PDF

**Other Supplementary Material for this manuscript includes the following:**

- Data file S1 (.csv format). qPCR array data.
- Data file S2 (.csv format). List of samples.
- Data file S3 (.csv format). List of assays.

**Files in this Data Supplement:**

- Adobe PDF - aau9124\_SM.pdf
