# Supplementary material for: Antibiotic resistance in European wastewater treatment plants mirrors the pattern of clinical antibiotic resistance prevalence
Source: Sci Adv. 2019 Mar 27;5(3):eaau9124. doi: 10.1126/sciadv.aau9124 (PMC6436925; doi:10.1126/sciadv.aau9124)
Supplement: Download PDF [file aau9124_SM.pdf]

Science Advances
AAAS

advances.sciencemag.org/cgi/content/full/5/3/eaau9124/DC1

Supplementary Materials for

Antibiotic resistance in European wastewater treatment plants mirrors the pattern of clinical antibiotic resistance prevalence

Katariina M. M. Pärnänen, Carlos Narciso-da-Rocha, David Kneis, Thomas U. Berendonk, Damiano Cacace, Thi Thuy Do, Christian Elpers, Despo Fatta-Kassinos, Isabel Henriques, Thomas Jaeger, Antti Karkman, Jose Luis Martinez, Stella G. Michael, Irene Michael-Kordatou, Kristin O'Sullivan, Sara Rodriguez-Mozaz, Thomas Schwartz, Hongjie Sheng, Henning Sørum, Robert D. Stedtfeld, James M. Tiedje, Saulo Varela Della Giustina, Fiona Walsh, Ivone Vaz-Moreira, Marko Virta*, Célia M. Manaia*

*Corresponding author. Email: cmanaia@porto.ucp.pt (C.M.M.); marko.virta@helsinki.fi (M.V.)

Published 27 March 2019, *Sci. Adv.* **5**, eaau9124 (2019)
DOI: 10.1126/sciadv.aau9124

The PDF file includes:

Fig. S1. Average richness values (number of positive assays) for the different influent and effluent wastewater samples from high (H_{AC}) and low (L_{AC}) antibiotic consumption countries, for resistance genes and mobile genetic elements.
Fig. S2. Food-producing animals' antibiotics consumption [expressed in biomass (mg/kg), values for 2013] and average maximal and minimal annual temperature and precipitation (yellow, average T_{min} >6°C; blue, average T_{max} <5°C).
Fig. S3. Average abundance (copies/ml; upper bars; left-hand legend) and prevalence values (gene copies/16*S* rRNA gene copies; lower bars; right-hand legend) for the different influent (Inf) and effluent (Eff) wastewater samples from high (H_{AC}) and low (L_{AC}) antibiotic consumption countries, determined on the basis of qPCR array for the genes: 16*S* rRNA, *intI1*, *bla*$_{OXA}$, *sul1*, *tetM*, *ermF*, *aadA*, *tnpA*, and *qacEdelta1*.
Fig. S4. Average abundance (copies/ml; upper bars; left-hand legend) and prevalence values (gene copies/16*S* rRNA copies; lower bars; right-hand legend) calculated by traditional real-time qPCR and qPCR array for Portuguese influent and effluent wastewater samples.
Table S1. Influent and effluent wastewater samples used in the study.
Table S2. qPCR primer sets and the percentage of samples that gave positive results for the influent and effluent wastewater samples.
Table S3. Consumption of antibacterials for systemic use (ATC group J01) in the community (primary care sector) in different European countries from 2005 to 2015 (see also Fig. 1), defined as daily dose per 1000 inhabitants and per day.
Table S4. Characterization of the UWTPs examined in this study, in terms of dimension, geographic conditions, treatment, and microbiological indicators.
Reference (*32*)

Other Supplementary Material for this manuscript includes the following:

(available at advances.sciencemag.org/cgi/content/full/5/3/eaau9124/DC1)

Data file S1. (.csv format). qPCR array data.
Data file S2. (.csv format). List of samples.
Data file S3. (.csv format). List of assays.

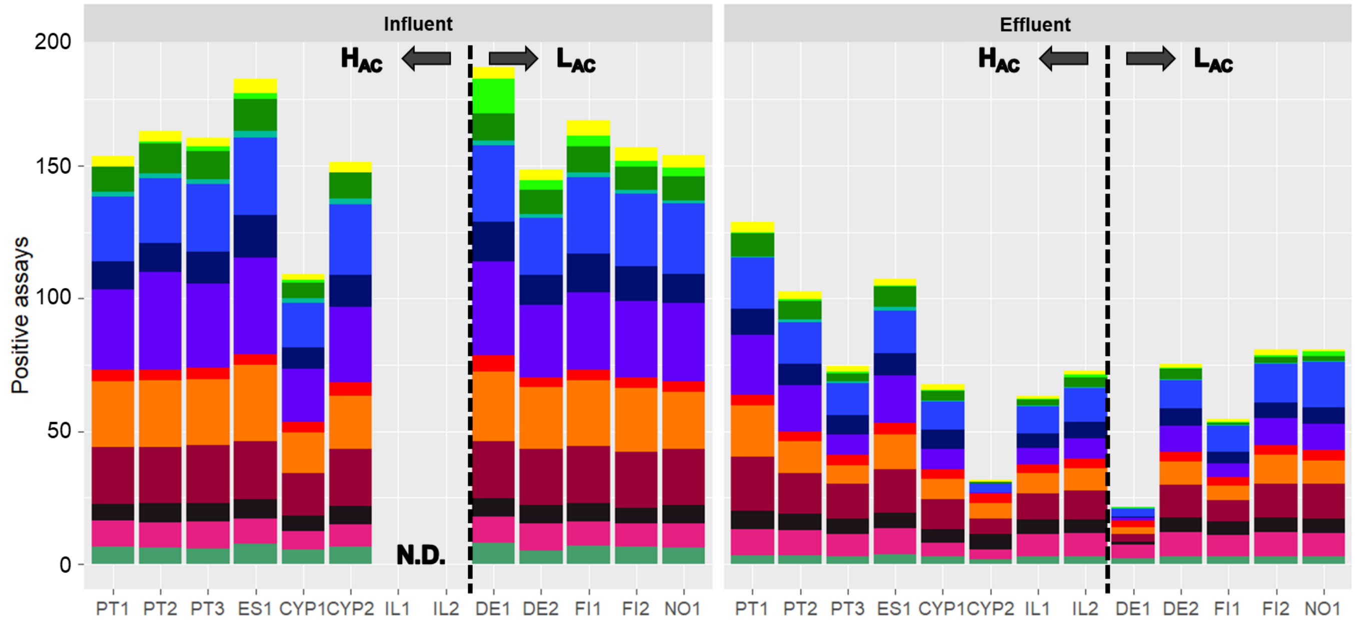

	Influent	Effluent
Other		
VAN	L_{AC}	
AMP		
QUI		
TET		
MLS_B		
BL		
SUL		
MDR		
AMG		
Integrase		
Transposase		
Insertional sequence		

Fig. S1. Average richness values (number of positive assays) for the different influent and effluent wastewater samples from high (H_{AC}) and low (L_{AC}) antibiotic consumption countries, for resistance genes and mobile genetic elements. In the legend, for each gene class are indicated the countries, H_{AC} or L_{AC}, with significantly higher richness ($p < 0.01$, Mann-Whitney U test). Resistance categories: AMG (aminoglycosides), MDR (multidrug resistance), SUL (sulphonamides), BL (beta-lactams), MLS_B (macrolide-lincosamide-streptogramin B), TET (tetracyclines), QUI (quinolones), AMP (amphenicols), VAN (vancomycin).

N.D. not determined, due to restrictions on influent wastewater sample collection in those UWTPs.

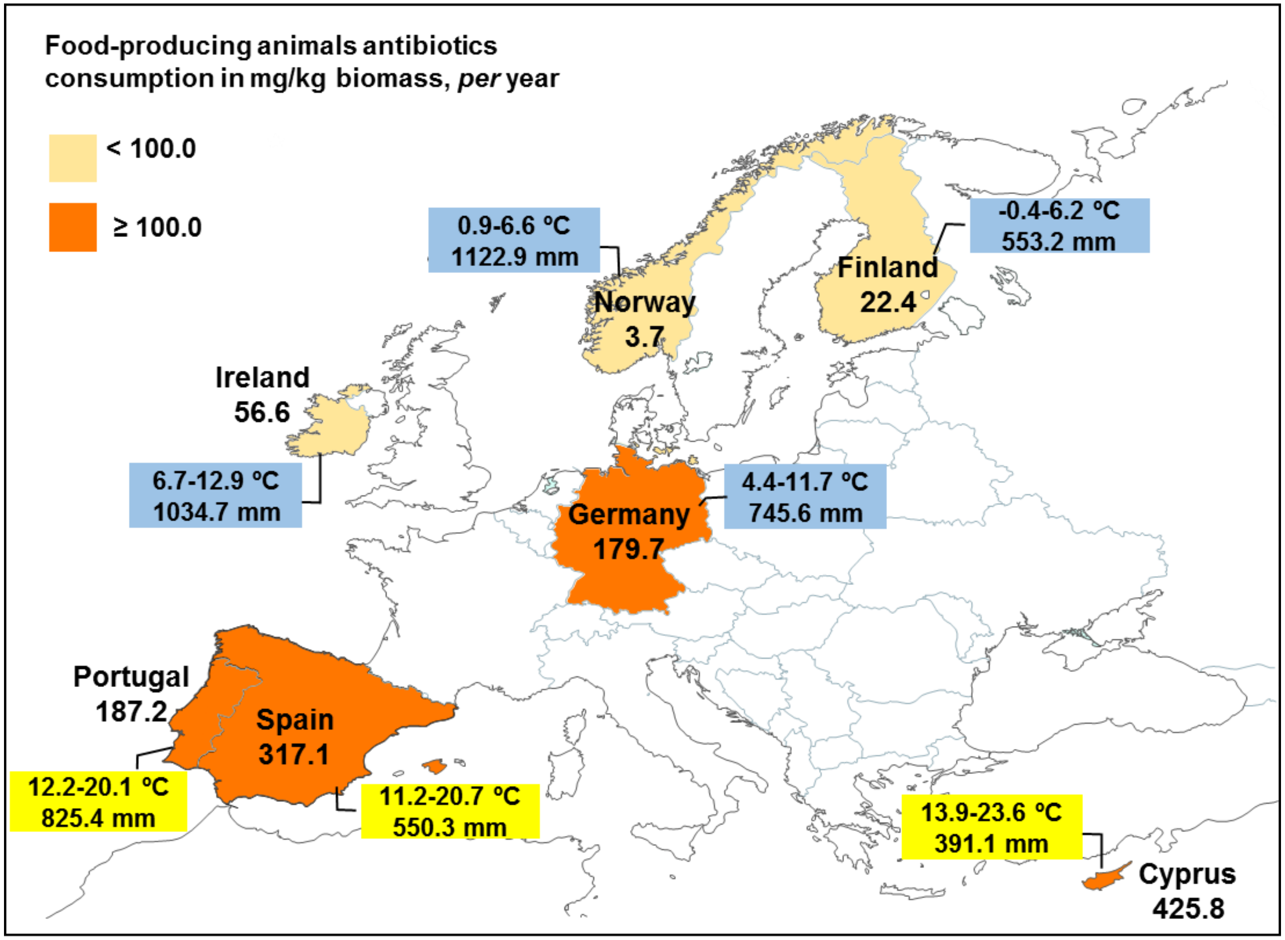

Fig. S2. Food-producing animals' antibiotics consumption [expressed in biomass (mg/kg), values for 2013] and average maximal and minimal annual temperature and precipitation (yellow, average T_{min} >6°C; blue, average T_{max} <5°C). Sources: Scientific report of ECDC, EFSA and EMA, 2017 (*32*) and weather database (http://www.weatherbase.com).

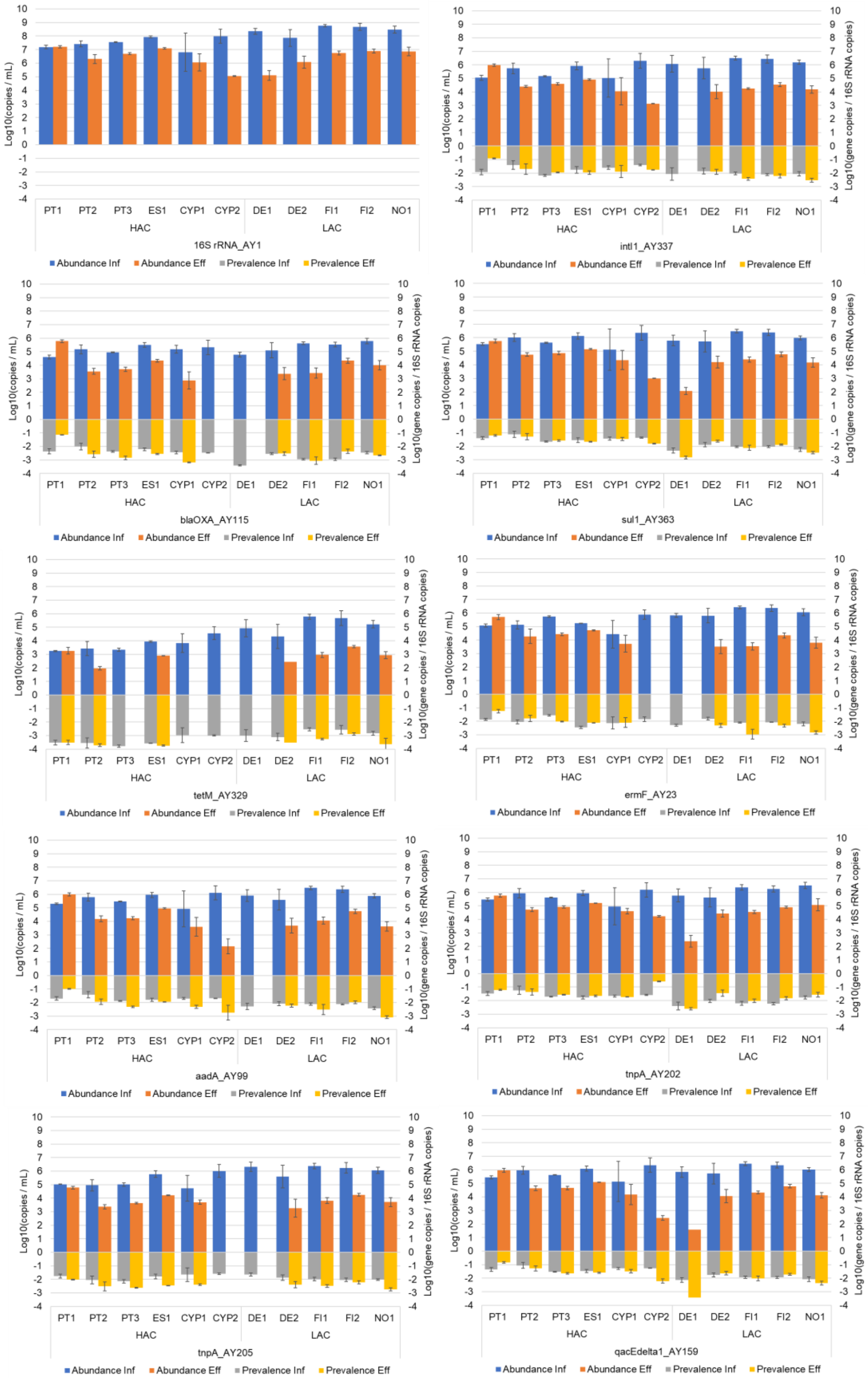
16S rRNA_AY1
intI1_AY337
blaOXA_AY115
sul1_AY363
tetM_AY329
ermF_AY23
aadA_AY99
tnpA_AY202
tnpA_AY205
qacEdelta1_AY159
Log10(copies / mL)
Log10(gene copies / 16S rRNA copies)
PT1
PT2
PT3
ES1
CYP1
CYP2
DE1
DE2
FI1
FI2
NO1
HAC
LAC
Abundance Inf
Abundance Eff
Prevalence Inf
Prevalence Eff

Fig. S3. Average abundance (copies/ml; upper bars; left-hand legend) and prevalence values (gene copies/16*S* rRNA gene copies; lower bars; right-hand legend) for the different influent (Inf) and effluent (Eff) wastewater samples from high (H_{AC}) and low (L_{AC}) antibiotic consumption countries, determined on the basis of qPCR array for the genes: 16*S* rRNA, *intI1*, *bla*$_{OXA}$, *sul1*, *tetM*, *ermF*, *aadA*, *tnpA*, and *qacEdelta1*. Error bars indicate the standard deviation.

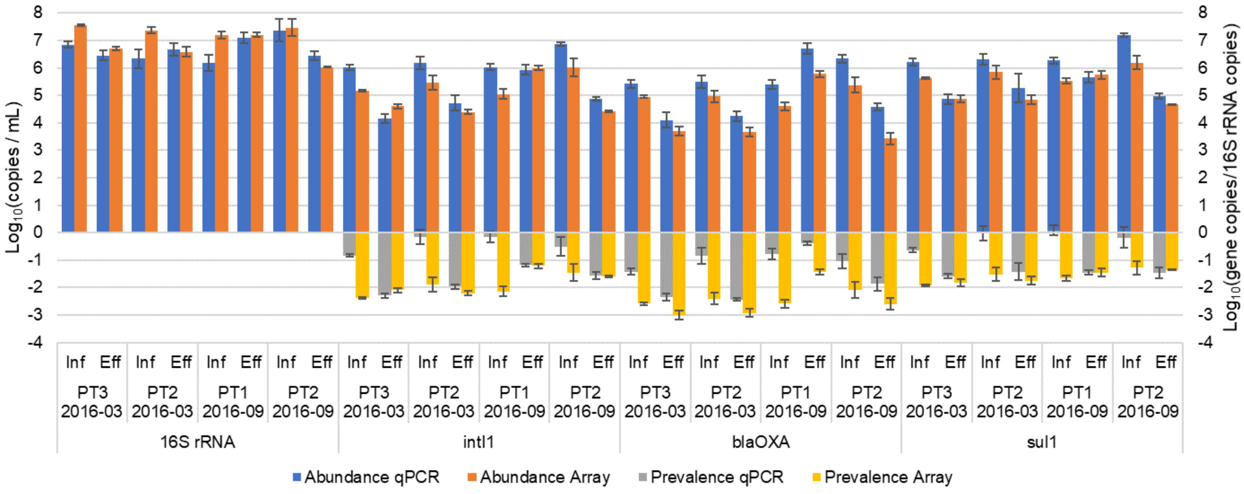

Fig. S4. Average abundance (copies/ml; upper bars; left-hand legend) and prevalence values (gene copies/16*S* rRNA copies; lower bars; right-hand legend) calculated by traditional real-time qPCR and qPCR array for Portuguese influent and effluent wastewater samples. Error bars indicate the standard deviation.

Table S1. Influent and effluent wastewater samples used in the study. (Between parentheses the number of days/samples that fulfilled the quality criteria to be analyzed by qPCR array)

Urban Wastewater Treatment Plant (UWTP) location		October 2015	March 2016		September 2016	
		Effluent	Influent	Effluent	Influent	Effluent
Portugal (PT) (North Portugal)	PT1	x (3d)	n.s.*	n.s.*	x (3d)	x (3d)
	PT2	x (3d)	x (3d)	x (3d)	x (3d)	x (3d)
	PT3	n.s.	x (3d)	x (3d)	n.s.	n.s.
Spain (ES) (Catalonia)	ES1	x (2d)	x (2d)	x (2d)	x (1d)	x (1d)
Cyprus (CYP) (Limassol and Nicosia)	CYP1	x (3d)	x (2d)	x (3d)	x (3d)	x (3d)
	CYP2	x (0d)	x (0d)	x (0d)	x (2d)	x (3d)
Ireland (IL) (no additional location available)	IL1	x (3d)	n.s.**	x (3d)	n.s.**	x (3d)
	IL2	x (3d)	n.s.**	x (3d)	n.s.**	x (3d)
Germany (DE) (Baden-Württemberg and Saxony)	DE1	x (1d)	x (3d)	x (0d)	x (3d)	x (2d)
	DE2	x (3d)	x (1d)	x (3d)	x (3d)	x (3d)
Finland (FI) (Uusimaa)	FI1	x (3d)	x (3d)	x (3d)	x (3d)	x (3d)
	FI2	x (3d)	x (3d)	x (3d)	x (3d)	x (3d)
Norway (NO) (Eastern Norway)	NO1	x (3d)	x (3d)	x (3d)	x (3d)	x (3d)

n.s. not sampled in these dates

*Since PT1 suffered an overflow during winter 2016 and was not operating in March 2016, samples were collected in another UWTP in the same region (PT3).

** The analysis of wastewater influent was not possible in Ireland due to restrictions on wastewater sample collection.

Table S2. qPCR primer sets and the percentage of samples that gave positive results for the influent and effluent wastewater samples. Primers are the same as in the original publication *(10)* except for those indicated with [a] which were added for this study

Functional Classification	Gene identification	Forward Primer	Reverse Primer	Assay	% Positive Influent	% Positive Effluent
**Housekeeping genes (HK)**						
	16S rRNA	GGGTTGCGCTCGTTGC	ATGGYTGTCGTCAGCTCGTG	AY1	100.0	100.0
	16S rRNA (2)[a]	CCTACGGGAGGCAGCAG	ATTACCGCGGCTGCTGGC	AY2	100.0	100.0
	gapA	CCGTTGAAGTGAAAGACGGTC	AACCACTTTCTTCGCACCAGC	AY347	96.0	31.5
	mdh	AAGAAACGGGCGTACTGACC	GTGGCTGATCTGACCAAACG	AY346	94.0	34.8
	rpoB	CGAACATCGGTCTGATCAACTC	GTTGCATGTTCGCACCCAT	AY345	44.0	18.5
	uidA	AACCACGCGTCTGTTGACTG	CCCGGTTGCCAGAGGTG	AY75	78.0	19.6
**Aminoglycosides (AMG)**						
deactivate	aac3-VI	CGTCACTTATTCGATGCCCTTAC	GTCGGGCGCGGCATA	AY93	34.0	23.9
deactivate	aac(6)I1	GACCGGATTAAGGCCGATG	CTTGCCTTGATATTCAGTTTTTATAACCA	AY5	0.0	0.0
deactivate	aac(6)-II	CGACCCGACTCCGAACAA	GCACGAATCCTGCCTTCTCA	AY8	84.0	20.7
deactivate	aac(6)-Ib	GTTTGAGAGGCAAGGTACCGTAA	GAATGCCTGGCGTGTTTGA	AY94	96.0	70.7
deactivate	aac(6)-Ib	CGTCGCCGAGCAACTTG	CGGTACCTTGCCTCTCAAACC	AY95	96.0	64.1
deactivate	aac(6)-Ib	AGAAGCACGCCCGACACTT	GCTCTCCATTCAGCATTGCA	AY172	98.0	67.4
deactivate	aac(6)-Iy	GCTTTGCGGATGCCTCAAT	GGAGAACAAAAATACCTTCAAGGAAA	AY7	0.0	0.0
deactivate	aadA	AGCTAAGCGCGAACTGCAAT	TGGCTCGAAGATACCTGCAA	AY96	100.0	98.9
deactivate	aadA	ACGGCTCCGCAGTGGAT	GGCCACAGTAACCAACAAATCA	AY97	100.0	87.0
deactivate	aadA	GTTGTGCACGACGACATCATT	GGCTCGAAGATACCTGCAAGAA	AY99	100.0	97.8
deactivate	aadA	CTTGTCGTGCATGACGACATC	TCGAAGATACCCGCAAGAATG	AY101	98.0	88.0
deactivate	aadA	CAATGACATTCTTGCGGGTATC	GACCTACCAAGGCAACGCTATG	AY167	100.0	98.9
deactivate	aadA	CGAGATTCTCCGCGCTGTA	GCTGCCATTCTCCAAATTGC	AY169	100.0	96.7
deactivate	aadA5	ATCACGATCTTGCGATTTTGCT	CTGCGGATGGGCCTAGAAG	AY98	96.0	67.4
deactivate	aadA5	GTTCTTGCTCTTGCTCGCATT	GATGCTCGGCAGGCAAAC	AY100	98.0	71.7
deactivate	aadA9	CGCGGCAAGCCTATCTTG	CAAATCAGCGACCGCAGACT	AY168	0.0	0.0
deactivate	aadA9	GGATGCACGCTTGGATGAA	CCTCTAGCGGCCGGAGTATT	AY173	0.0	0.0

deactivate	aadD	CCGACAACATTTCTACCATCCTT	ACCGAAGCGCTCGTCGTATA	AY151	52.0	8.7
deactivate	aadE	TACCTTATTGCCCTTGGAAGAGTTA	GGAACTATGTCCCTTTTAATTCTACAATCT	AY174	96.0	28.3
deactivate	acc	CCCTGCGTTGTGGCTATGT	TTGGCCACGCCAATCC	AY382	0.0	0.0
deactivate	aph	TTTCAGCAAGTGGATCATGTTAAAAT	CCAAGCTGTTTCCACTGTTTTTC	AY166	0.0	0.0
deactivate	aphA1/7	TGAACAAGTCTGGAAAGAAATGCA	CCTATTAATTTCCCCTCGTCAAAAA	AY170	96.0	38.0
deactivate	aphA3	AAAAGCCCGAAGAGGAACTTG	CATCTTTCACAAAGATGTTGCTGTCT	AY14	96.0	18.5
deactivate	aphA3	CGGAATTGAAAAAACTGATCGAA	ATACCGGCTGTCCGTCATTT	AY171	96.0	35.9
deactivate	aph(2)-Ib	TGAGCAGTATCATAAGTTGAGTGAAAAG	GACAGAACAATCAATCTCTATGGAATG	AY104	60.0	0.0
deactivate	aph(2)-Id	TAAGGATATACCGACAGTTTTGGAAA	TTTAATCCCTCTTCATACCAATCCATA	AY105	6.0	0.0
deactivate	aph6	CCCATCCCATGTGTAAGGAAA	GCCACCGCTTCTGCTGTAC	AY15	0.0	0.0
deactivate	sat4	GAATGGGCAAAGCATAAAAACTTG	CCGATTTTGAAACCACAATTATGATA	AY49	96.0	16.3
deactivate	spcN	AAAAGTTCGATGAAACACGCCTAT	TCCAGTGGTAGTCCCCGAATC	AY286	0.0	0.0
deactivate	spcN	CAGAATCTTCCTGAAAAGTTTGATGAA	CGCAGACACGCCGAATC	AY287	0.0	0.0
deactivate	str	AATGAGTTTTGGAGTGTCTCAACGTA	AATCAAAACCCCTATTAAAGCCAAT	AY175	90.0	2.2
deactivate	strA	CCGGTGGCATTTGAGAAAAA	GTGGCTCAACCTGCGAAAAG	AY176	90.0	10.9
deactivate	strB	GCTCGGTCGTGAGAACAATCT	CAATTTCGGTCGCCTGGTAGT	AY177	100.0	95.7
**Amphenicols (AMP)**						
deactivate	catA1	GGGTGAGTTTCACCAGTTTTGATT	CACCTTGTCGCCTTGCGTATA	AY130	94.0	4.3
deactivate	catB3	GCACTCGATGCCTTCCAAAA	AGAGCCGATCCAAACGTCAT	AY51	96.0	41.3
deactivate	catB8	CACTCGACGCCTTCCAAAG	CCGAGCCTATCCAGACATCATT	AY52	86.0	17.4
efflux	ceoA	ATCAACACGGACCAGGACAAG	GGAAAGTCCGCTCACGATGA	AY53	0.0	0.0
efflux	cmlA	AATTTTGCCGATTATTGCTGAAA	GATTGTCATCATTCGTTTATCACCAA	AY20	0.0	0.0
efflux	cmlA	TAGGAAGCATCGGAACGTTGAT	CAGACCGAGCACGACTGTTG	AY127	96.0	43.5
efflux	cmlA	AGGAAGCATCGGAACGTTGA	ACAGACCGAGCACGACTGTTG	AY128	56.0	40.2
efflux	cmlA	GCGCTCTTCGAGGATTCG	CCGCCCAAGCAGAAGTAGAC	AY375	96.0	43.5
efflux	cmxA	GCGATCGCCATCCTCTGT	TCGACACGGAGCCTTGGT	AY129	86.0	35.9
efflux	floR	ATTGTCTTCACGGTGTCCGTTA	CCGCGATGTCGTCGAACT	AY56	92.0	44.6
efflux	floR	TCGTCATCTACGGCCTTTTC	CTTGACTTGATCCAGAGGGC	AY335	8.0	0.0
efflux	mdtL	TGCTGATCGGGATTCTGATTG	CAGGCGCGACGAACATAAT	AY88	96.0	33.7
efflux	rarD	GCGGGTGTGGTCACTACGAT	AGCGTTGGGCCGATATACTG	AY131	28.0	1.1

efflux	rarD	TGACGCATCGCGTGATCT	AAATTTTCTGTGGCGTCTGAATC	AY132	96.0	29.3
efflux	yidY/mdtL	GCAGTTGCATATCGCCTTCTC	CTTCCCGGCAAACAGCAT	AY87	24.0	5.4
**Beta-lactams (BL)**						
deactivate	ampC	GCAGCACGCCCCGTAA	TGTACCCATGATGCGCGTACT	AY30	86.0	23.9
deactivate	ampC	AACAAAAGATCCCCGGTATGG	ACGCCCGTAAATGTTTTGCT	AY31	96.0	43.5
deactivate	ampC	TCCGGTGACGCGACAGA	CAGCACGCCGGTGAAAGT	AY33	2.0	0.0
deactivate	ampC	CCGCCCAGAGCAAGGACTA	GCTCGACTTCACGCCGTAAG	AY114	0.0	0.0
deactivate	ampC	GCAGCGAAGCGTCAGTCA	AGATCCGTGGCCGCATAA	AY258	4.0	0.0
deactivate	bla1	GCAAGTTGAAGCGAAAGAAAAGA	TACCAGTATCAATCGCATATACACCTAA	AY39	0.0	0.0
deactivate	blaACC	CACACAGCTGATGGCTTATCTAAAA	AATAAACGCGATGGGTTCCA	AY28	0.0	0.0
deactivate	blaACT	TGGCGTATCGGGTCAATGT	CTCCACGGGCCAGTTGAG	AY29	12.0	0.0
deactivate	blaACT	CTGTTCGAGCTGGGTTCTATAAGTAAA	CAGTATCTGGTCACCGGATCGT	AY109	46.0	0.0
deactivate	blaCMY	CCGCGGCGAAATTAAGC	GCCACTGTTTGCCTGTCAGTT	AY32	88.0	12.0
deactivate	blaCMY	AAAGCCTCATGGGTGCATAAA	ATAGCTTTTGTTTGCCAGCATCA	AY108	94.0	16.3
deactivate	blaCMY	GCGAGCAGCCTGAAGCA	CGGATGGGCTTGTCCTCTT	AY111	84.0	20.7
deactivate	blaCTX-M	GGAGGCGTGACGGCTTTT	TTCAGTGCGATCCAGACGAA	AY116	34.0	0.0
deactivate	blaCTX-M	GCCGCGGTGCTGAAGA	ATCGGATTATAGTTAACCAGGTCAGATTT	AY117	74.0	8.7
deactivate	blaCTX-M	CGATACCACCACGCCGTTA	GCATTGCCCAACGTCAGATT	AY118	8.0	0.0
deactivate	blaCTX-M	CTTGGCGTTGCGCTGAT	CGTTCATCGGCACGGTAGA	AY119	18.0	6.5
deactivate	blaCTX-M	GCGATAACGTGGCGATGAAT	GTCGAGACGGAACGTTTCGT	AY162	76.0	3.3
deactivate	blaCTX-M	CACAGTTGGTGACGTGGCTTAA	CTCCGCTGCCGGTTTTATC	AY165	38.0	3.3
deactivate	blaCTX-M	CGTCACGCTGTTGTTAGGAA	CGCTCATCAGCACGATAAAG	AY324	84.0	7.6
deactivate	blaCTX-M[a]	CGATGTGCAGTACCAGTAA	GCAATGGGATTGTAGTTAA	AY326	16.0	1.1
deactivate	blaCTX-M[a]	CTATGGCACCACCAACGATA	ACGGCTTTCTGCCTTAGGTT	AY360	84.0	7.6
deactivate	blaDHA	TGGCCGCAGCAGAAAGA	CCGTTTTATGCACCCAGGAA	AY112	14.0	0.0
deactivate	blaFOX	GGTTTGCCGCTGCAGTTC	GCGGCCAGGTGACCAA	AY113	38.0	45.7
deactivate	blaGES	GCAATGTGCTCAACGTTCAAG	GTGCCTGAGTCAATTCTTTCAAAG	AY120	100.0	56.5
deactivate	blaIMP	AACACGGTTTGGTGGTTCTTGTA	GCGCTCCACAAACCAATTG	AY148	12.0	19.6
deactivate	blaIMP	AAGGCAGCATTTCCTCTCATTTT	GGATAGATCGAGAATTAAGCCACTCT	AY242	8.0	46.7
deactivate	blaIMP	GGAATAGAGTGGCTTAATTC	GGTTTAACAAAACAACCACC	AY327	6.0	23.9

deactivate	blaKPC	CAGCTCATTCAAGGGCTTTC	GGCGGCGTTATCACTGTATT	AY323	14.0	0.0
deactivate	blaKPC	GCCGCCGTGCAATACAGT	GCCGCCCAACTCCTTCA	AY367	16.0	2.2
deactivate	blaL1	CACCGGGTTACCAGCTGAAG	GCGAAGCTGCGCTTGTAGTC	AY48	0.0	1.1
deactivate	blaMOX/blaCMY	CTATGTCAATGTGCCGAAGCA	GGCTTGTCCTCTTTCGAATAGC	AY34	94.0	21.7
deactivate	blaNDM	GGCCACACCAGTGACAATATCA	CAGGCAGCCACCAAAAGC	AY362	0.0	0.0
deactivate	blaOCH	GGCGACTTGCGCCGTAT	TTTTCTGCTCGGCCATGAG	AY35	2.0	0.0
deactivate	blaOKP	GCCGCCATCACCATGAG	GGTGACGTTGTCACCGATCTG	AY40	6.0	0.0
deactivate	blaOXA	CGCAATTATCGGCCTAGAAACT	TTGGCTTTCCGTCCCATTT	AY44	100.0	90.2
deactivate	blaOXA	CGGATGGTTTGAAGGGTTTATTAT	TCTTGGCTTTTATGCTTGATGTTAA	AY115	96.0	83.7
deactivate	blaOXA	GCAATTGCCTTTTAAACCTGA	CTGCCTTTTCAACAAAACCC	AY322	96.0	35.9
deactivate	blaOXA[a]	CACTTACAGGAAACTTGGGGTCG	AGTGTGTTTAGAATGGTGATC	AY361	96.0	78.3
deactivate	blaOXY	CGTTCAGGCGGCAGGTT	GCCGCGATATAAGATTTGAGAATT	AY42	18.0	0.0
deactivate	blaPDC	CGCCGTACAACCGGTGAT	GAAGTAATGCGGTTCTCCTTTCA	AY36	2.0	0.0
deactivate	blaPER	TGCTGGTTGCTGTTTTTGTGA	CCTGCGCAATGATAGCTTCAT	AY241	80.0	9.8
deactivate	blaPSE	TTGTGACCTATTCCCCTGTAATAGAA	TGCGAAGCACGCATCATC	AY43	70.0	10.9
deactivate	blaROB	GCAAAGGCATGACGATTGC	CGCGCTGTTGTCGCTAAA	AY41	10.0	1.1
deactivate	blaSFO	CCGCCGCCATCCAGTA	GGGCCGCCAAGATGCT	AY121	64.0	6.5
deactivate	blaSHV	TCCCATGATGAGCACCTTTAAA	TTCGTCACCGGCATCCA	AY37	96.0	6.5
deactivate	blaSHV	CTTTCCCATGATGAGCACCTTT	TCCTGCTGGCGATAGTGGAT	AY163	90.0	4.3
deactivate	blaSHV[a]	CGCTTTCCCATGATGAGCACCTTT	TCCTGCTGGCGATAGTGGATCTTT	AY272	58.0	3.3
deactivate	blaSHV	GCGTTATTTTCGCCTGTGTA	AGGTGCTCATCATGGGAAAG	AY325	76.0	0.0
deactivate	blaSRT	CAGCCGCTGATGAAAAAATATG	CAGCGAGCCCACTTCGA	AY259	0.0	0.0
deactivate	blaTEM[a]	TTCCTGTTTTTGCTCACCCAG	CTCAAGGATCTTACCGCTGTTG	AY3	94.0	45.7
deactivate	blaTEM	AGCATCTTACGGATGGCATGA	TCCTCCGATCGTTGTCAGAAGT	AY164	96.0	53.3
deactivate	blaTLA	ACACTTTGCCATTGCTGTTTATGT	TGCAAATTTCGGCAATAATCTTT	AY122	0.0	0.0
deactivate	blaVEB	CCCGATGCAAAGCGTTATG	GAAAGATTCCCTTTATCTATCTCAGACAA	AY38	100.0	45.7
deactivate	blaVIM	GCACTTCTCGCGGAGATTG	CGACGGTGATGCGTACGTT	AY147	36.0	0.0
deactivate	blaVIM2[a]	CCTCCATTGAGCGGATTCA	GCCGTGCCCCGGAA	AY260	10.0	0.0
deactivate	blaZ	GGAGATAAAGTAACAAATCCAGTTAGATATGA	TGCTTAATTTTCCATTTGCGATAAG	AY123	2.0	0.0
deactivate	ccrA	GCAGCGTTGCTGGACACA	GTTCGGGATAAACGTGGTGACT	AY24	22.0	0.0

deactivate	cepA	AGTTGCGCAGAACAGTCCTCTT	TCGTATCTTGCCCGTCGATAAT	AY107	82.0	18.5
deactivate	cfxA	TCATTCCTCGTTCAAGTTTTCAGA	TGCAGCACCAAGAGGAGATGT	AY106	98.0	84.8
deactivate	cphA	GCGAGCTGCACAAGCTGAT	CGGCCCAGTCGCTCTTC	AY46	90.0	22.8
deactivate	cphA	GTGCTGATGGCGAGTTTCTG	GGTGTGGTAGTTGGTGTTGATCAC	AY47	92.0	21.7
protection	mecA	GGTTACGGACAAGGTGAAATACTGAT	TGTCTTTTAATAAGTGAGGTGCGTTAATA	AY155	0.0	0.0
protection	mecA[a]	GTTGTAGTTGTCGGGTTTGG	CTTCCACATACCATCTTCTTTAAC	AY284	0.0	3.3
protection	pbp	CCGGTGCCATTGGTTTAGA	AAAATAGCCGCCCCAAGATT	AY154	14.0	0.0
protection	pbp2x	TTTCATAAGTATCTGGACATGGAAGAA	CCAAAGGAAACTTGCTTGAGATTAG	AY237	0.0	0.0
protection	pbp5	GGCGAACTTCTAATTAATCCTATCCA	CGCCGATGACATTCTTCTTATCTT	AY153	24.0	0.0
protection	penA	AGACGGTAACGTATAACTTTTTGAAAGA	GCGTGTAGCCGGCAATG	AY236	0.0	0.0
deactivate	sdeB	GGCATGCAGAAAGTGTTTATGC	TTAAGTGCGGAAGCGGTAGTG	AY353	0.0	0.0
**Quinolones (QUI)**						
efflux	pmrA	TTTGCAGGTTTTGTTCCTAATGC	GCAGAGCCTGATTTCTCCTTTG	AY239	0.0	0.0
protection	qnrA	AGGATTTCTCACGCCAGGATT	CCGCTTTCAATGAAACTGCAA	AY248	14.0	1.1
protection	qnrB	GCGACGTTCAGTGGTTCAGA	GCTGCTCGCCAGTCGAA	AY328	58.0	5.4
protection	qnrSrtF11[a]	GACGTGCTAACTTGCGTGAT	TGGCATTGTTGGAAACTTG	AY6	96.0	56.5
**Multidrug Resistance (MDR)**						
efflux	acrA	CAACGATCGGACGGGTTTC	TGGCGATGCCACCGTACT	AY61	92.0	8.7
efflux	acrA	GGTCTATCACCCTACGCGCTATC	GCGCGCACGAACATACC	AY62	80.0	12.0
efflux	acrA	CAGACCCGCATCGCATATT	CGACAATTTCGCGCTCATG	AY249	0.0	0.0
efflux	acrA	TACTTTGCGCGCCATCTTC	CGTGCGCGAACGAACAT	AY256	28.0	2.2
efflux	acrA	CGTGCGCGAACGAACA	ACTTTGCGCGCCATCTTC	AY257	50.0	13.0
efflux	acrB	AGTCGGTGTTCGCCGTTAAC	CAAGGAAACGAACGCAATACC	AY9	96.0	23.9
efflux	acrB	TGGTAGTGGGCGTCATTAACAC	GGCAACGTAATCCGAAATATCC	AY10	0.0	0.0
efflux	acrF	GCGGCCAGGCACAAAA	TACGCTCTTCCCACGGTTTC	AY11	92.0	20.7
regulator	acrR	GCGCTGGAGACACGACAAC	GCCTTGCTGCGAGAACAAA	AY102	76.0	2.2
regulator	acrR	GATGATACCCCCTGCTGTGAGA	ACCAAACAAGAAGCGCAAGAA	AY103	96.0	34.8
regulator	acrR	TGCAACACGCGCTTTCTC	ACGATTGCGGGCAGGTT	AY350	22.0	17.4
efflux	adeA	CAGTTCGAGCGCCTATTTCTG	CGCCCTGACCGACCAAT	AY12	18.0	0.0
efflux	bexA	GCGGATCTCTGGTCAGCAA	TGATTGATGGTTCCCCGTACA	AY27	84.0	15.2

protection	cfr	GCAAAATTCAGAGCAAGTTACGAA	AAAATGACTCCCAACCTGCTTTAT	AY277	0.0	0.0
efflux	cmeA	GCAGCAAAGAAGAAGCACCAA	AGCAGGGTAAGTAAAACTAAGTGGTAAATCT	AY50	0.0	0.0
efflux	cmr	CGGCATCGTCAGTGGAATT	CGGTTCCGAAAAAGATGGAA	AY57	0.0	0.0
efflux	emrB/qacA	CTTTTCTCTAACCGTACATTATCTACGATAAA	AGAACGTAGCGACTGATAAAATGCT	AY156	0.0	0.0
efflux	emrB/qacA	GCAGTAGAAGGAACGATTGTTAGTACAG	TGCGTAAACCCAGCTAACAAGTT	AY352	0.0	0.0
efflux	emrD	CTCAGCAGTATGGTGGTAAGCATT	ACCAGGCGCCGAAGAAC	AY64	16.0	0.0
regulator	marR	GCGGCGTACTGGTGAAGCTA	TGCCCTGGTCGTTGATGA	AY254	0.0	0.0
regulator	marR	TCTGGCGTTAGCTTCACCAGTAC	GTGCAAAGGCTGGATCGAA	AY255	0.0	0.0
regulator	marR	GCTGTTGATGACATTGCTCACA	CGGCGTACTGGTGAAGCTAAC	AY355	12.0	0.0
efflux	mdsA	CGGAGTCCATCGACCATTTG	ATCGTCGGCAAGGAGAATCA	AY253	0.0	0.0
efflux	mdtA	CCTAACGGGCGTGACTTCA	TTCACCTGTTTCAAGGGTCAAA	AY78	0.0	0.0
efflux	mdtE	CGTCGGCGCACTCGTT	TCCAGACGTTGTACGGTAACCA	AY81	84.0	16.3
efflux	mdtF	CCACCATCGGGCTTTCC	CCCTTCTTTCTGCATCATCTCA	AY373	96.0	32.6
efflux	mdtG	TGGCACAAAATATCTGGCAGTT	TTGTGTGGCGATAAGAGCATTAG	AY82	96.0	31.5
efflux	mdtG	TTATCTGTTTTCTGCTCACCTTCTTTT	GCGTGGTGACAAACAGGCTTA	AY83	0.0	0.0
efflux	mdtH	CGCGTGAAACCTTAAGTGCTT	AGACGGCTAAACCCCATATAGCT	AY85	96.0	26.1
efflux	mdtH	CTGCCGTTAAATGGATGTATGC	ACTCCAGCGGGCGATAGG	AY86	94.0	33.7
efflux	mepA	ATCGGTCGCTCTTCGTTCAC	ATAAATAGGATCGAGCTGCTGGAT	AY245	8.0	5.4
efflux	mexA	AGGACAACGCTATGCAACGAA	CCGGAAAGGGCCGAAAT	AY89	4.0	0.0
efflux	mexB	CTGGAGATCGACGACGAGAAG	GAAATCGTTGACGTAGCTGGAA	AY374	6.0	0.0
efflux	mexD	TTGCCACTGGCTTTCATGAG	CACTGCGGAGAACTGTCTGTAGA	AY90	0.0	0.0
efflux	mexE	GGTCAGCACCGACAAGGTCTAC	AGCTCGACGTACTTGAGGAACAC	AY246	90.0	20.7
efflux	mexF	CCGCGAGAAGGCCAAGA	TTGAGTTCGGCGGTGATGA	AY247	46.0	37.0
efflux	mtrC	GGACGGGAAGATGGTCCAA	CGTAGCGTTCCGGTTCGAT	AY250	0.0	0.0
efflux	mtrD	TGCGCGTAGTCGTTCATCTC	CGTTCCAATTTCCTGATGATTG	AY230	0.0	0.0
efflux	mtrD	GGTCGGCACGCTCTTGTC	TGAAGAATTTGCGCACCACTAC	AY232	24.0	0.0
efflux	mtrD	CCGCCAAGCCGATATAGACA	GGCCGGGTTGCCAAA	AY233	0.0	2.2
efflux	mtrE	CGATGTGTCGTTTTGGAAGGT	CCTGCACCATGATTCCTCAATA	AY231	0.0	0.0
efflux	oprD	ATGAAGTGGAGCGCCATTG	GGCCACGGCGAACTGA	AY234	34.0	17.4
efflux	oprJ	ACGAGAGTGGCGTCGACAA	AAGGCGATCTCGTTGAGGAA	AY235	44.0	23.9

efflux	qac	GGAGATTTAGCTCATGTAGCTGAAGAA	AAGCTGTTTTATCCCCGTAGCTTTA	AY356	4.0	1.1
efflux	qacA/qacB	TGGCAATAGGAGCTATGGTGTTT	AAGGTAACACTATTTTCGGTCCAAATC	AY262	2.0	0.0
efflux	qacEdelta1	TCGCAACATCCGCATTAAAA	ATGGATTTCAGAACCAGAGAAAGAAA	AY126	100.0	97.8
efflux	qacEdelta1	CCCCTTCCGCCGTTGT	CGACCAGACTGCATAAGCAACA	AY159	100.0	98.9
efflux	qacEdelta1	GTCGGTGTTGCTTATGCAGTCT	CAACCAGGCAATGGCTGTAA	AY351	100.0	100.0
efflux	qacG	CAATAATAACCGAAATAATAGGGACAAGTT	AATAAGTGTTCCTAGTGTTGGCCATAG	AY261	0.0	0.0
efflux	qacH	GTGGCAGCTATCGCTTGGAT	CCAACGAACGCCCACAA	AY124	88.0	47.8
efflux	qacH	CATCGTGCTTGTGGCAGCTA	TGAACGCCCAGAAGTCTAGTTTT	AY125	100.0	94.6
efflux	sdeB	CACTACCGCTTCCGCACTTAA	TGAAAAAACGGGAAAAGTCCAT	AY160	0.0	0.0
efflux	tolC	GGCCGAGAACCTGATGCA	AGACTTACGCAATTCCGGGTTA	AY298	96.0	32.6
efflux	tolC	CAGGCAGAGAACCTGATGCA	CGCAATTCCGGGTTGCT	AY299	68.0	3.3
efflux	tolC	GCCAGGCAGAGAACCTGATG	CGCAATTCCGGGTTGCT	AY300	66.0	6.5
efflux	ttgA	ACGCCAATGCCAAACGATT	GTCACGGCGCAGCTTGA	AY243	50.0	9.8
efflux	ttgB	TCGCCCTGGATGTACACCTT	ACCATTGCCGACATCAACAAC	AY244	90.0	34.8
efflux	yceL/mdtH	TCGGGATGGTGGGCAAT	CGATAACCGAGCCGATGTAGA	AY84	0.0	0.0
**Macrolide-Lincosamide-Streptogramin B (MLS$_B$)**						
efflux	carB	GGAGTGAGGCTGACCGTAGAAG	ATCGGCGAAACGCACAAA	AY288	0.0	0.0
deactivate	ereA	CCTGTGGTACGGAGAATTCATGT	ACCGCATTCGCTTTGCTT	AY60	100.0	58.7
deactivate	ereB	GCTTTATTTCAGGAGGCGGAAT	TTTTAAATGCCACAGCACAGAATC	AY63	72.0	4.3
protection	erm34	GCGCGTTGACGACGATTT	TGGTCATACTCGACGGCTAGAAC	AY134	0.0	0.0
protection	erm(35)	TTGAAAACGATGTTGCATTAAGTCA	TCTATAATCACAACTAACCACTTGAACGT	AY21	28.0	3.3
protection	erm(36)	GGCGGACCGACTTGCAT	TCTGCGTTGACGACGGTTAC	AY91	0.0	0.0
protection	ermA	TTGAGAAGGGATTTGCGAAAAG	ATATCCATCTCCACCATTAATAGTAAACC	AY274	2.0	2.2
protection	ermA/ermTR	ACATTTTACCAAGGAACTTGTGGAA	GTGGCATGACATAAACCTTCATCA	AY283	2.0	0.0
protection	ermB	TAAAGGGCATTTAACGACGAAACT	TTTATACCTCTGTTTGTTAGGGAATTGAA	AY136	96.0	82.6
protection	ermC	TTTGAAATCGGCTCAGGAAAA	ATGGTCTATTTCAATGGCAGTTACG	AY275	2.0	4.3
protection	ermD	GGACTCGGCAATGGTCAGAA	CCCCGAAACGCAATATAATGTT	AY18	0.0	0.0
protection	ermF	CAGCTTTGGTTGAACATTTACGAA	AAATTCCTAAAATCACAACCGACAA	AY23	100.0	94.6
protection	ermK	GTTTGATATTGGCATTGTCAGAGAAA	ACCATTGCCGAGTCCACTTT	AY17	0.0	0.0
protection	ermK	GAGCCGCAAGCCCC TTT	GTGTTTCATTTGACGCGGAGTAA	AY19	0.0	0.0

protection	ermT	GTTCACTAGCACTATTTTTAATGACAGAAGT	GAAGGGTGTCTTTTTAATACAATTAACGA	AY137	38.0	2.2
protection	ermT	GTAAAATCCCTAGAGAATACTTTCATCCA	TGAGTGATATTTTTGAAGGGTGTCTT	AY238	36.0	2.2
protection	ermX	GCTCAGTGGTCCCCATGGT	ATCCCCCCGTCAACGTTT	AY209	86.0	7.6
protection	ermY	TTGTCTTTGAAAGTGAAGCAACAGT	TAACGCTAGAGAACGATTTGTATTGAG	AY270	0.0	0.0
efflux	lmrA	TTCAGATGCAATGGCGTTTG	ATAATCGGGAACATAATGAGCATAACTAC	AY77	58.0	0.0
efflux	lmrA	TCGACGTGACCGTAGTGAACA	CGTGACTACCCAGGTGAGTTGA	AY252	0.0	0.0
deactivate	lnuA	TGACGCTCAACACACTCAAAAA	TTCATGCTTAAGTTCCATACGTGAA	AY251	24.0	0.0
deactivate	lnuB	TGAACATAATCCCCTCGTTTAAAGAT	TAATTGCCCTGTTTCATCGTAAATAA	AY67	96.0	62.0
deactivate	lnuB	AAAGGAGAAGGTGACCAATACTCTGA	GGAGCTACGTCAAACAACCAGTT	AY68	96.0	67.4
deactivate	lnuC	TGGTCAATATAACAGATGTAAACCAGATTT	CACCCCAGCCACCATCAA	AY278	0.0	3.3
efflux	matA/mel	TAGTAGGCAAGCTCGGTGTTGA	CCTGTGCTATTTTAAGCCTTGTTTCT	AY139	100.0	82.6
efflux	mefA	CCGTAGCATTGGAACAGCTTTT	AAACGGAGTATAAGAGTGCTGCAA	AY146	96.0	83.7
deactivate	mphA	CTGACGCGCTCCGTGTT	GGTGGTGCATGGCGATCT	AY140	66.0	22.8
deactivate	mphA	TGATGACCCTGCCATCGA	TTCGCGAGCCCCTCTTC	AY142	70.0	28.3
deactivate	mphB	CGCAGCGCTTGATCTTGTAG	TTACTGCATCCATACGCTGCTT	AY141	14.0	0.0
deactivate	mphC	CGTTTGAAGTACCGAATTGGAAA	GCTGCGGGTTTGCCTGTA	AY273	0.0	0.0
efflux	msrA	CTGCTAACACAAGTACGATTCCAAAT	TCAAGTAAAGTTGTCTTACCTACACCATT	AY149	0.0	0.0
efflux	msrA	AACGAAATCAAGCGCAACAA	CAACCGTGCCTTTTTCTTTTG	AY276	0.0	3.3
efflux	msrC	TCAGACCGGATCGGTTGTC	CCTATTTTTTGGAGTCTTCTCTCTAATGTT	AY138	22.0	0.0
efflux	msrC	GAATCACTTGTCCGCAGTTTGTT	CGTACACAACGGTTTCGTCAGA	AY150	0.0	0.0
efflux	oleC	CCCGGAGTCGATGTTCGA	GCCGAAGACGTACACGAACAG	AY285	0.0	3.3
protection	pikR1	TCGACATGCGTGACGAGATT	CCGCGAATTAGGCCAGAA	AY289	0.0	0.0
protection	pikR2	TCGTGGGCCAGGTGAAGA	TTCCCCTTGCCGGTGAA	AY290	0.0	0.0
protection	pncA	GCAATCGAGGCGGTGTTC	TTGCCGCAGCCAATTCA	AY229	2.0	5.4
deactivate	vatB	GGAAAAAGCAACTCCATCTCTTGA	TCCTGGCATAACAGTAACATTCTGA	AY224	4.0	0.0
deactivate	vatB	TTGGGAAAAAGCAACTCCATCT	CAATCCACACATCATTTCCAACA	AY266	4.0	0.0
deactivate	vatC	CGATGTTTGGATTGGACGAGAT	GCTGCAATAATAGCCCCGTTT	AY267	0.0	0.0
deactivate	vatC	CGGAAATTGGGAACGATGTT	GCAATAATAGCCCCGTTTCCTA	AY225	0.0	0.0
deactivate	vatD	TGCAATAGTAGCTGCTAATTCTGTTGTT	TGTTTTATTTCGTTAGCAGGATTTCC	AY73	0.0	0.0
deactivate	vatE	GGTGCCATTATCGGAGCAAAT	TTGGATTGCCACCGACAAT	AY74	0.0	0.0

deactivate	vatE	GACCGTCCTACCAGGCGTAA	TTGGATTGCCACCGACAATT	AY263	0.0	0.0
deactivate	vgb	AGGGAGGGTATCCATGCAGAT	ACCAAATGCGCCCGTTT	AY144	0.0	0.0
deactivate	vgb	CCACGATGGCTGCCTTTG	GGCCATGCAGGACGGATAT	AY145	0.0	0.0
efflux	vgaA	CGAGTATTGTGGAAAGCAGCTAGTT	CCCGTACCGTTAGAGCCGATA	AY226	0.0	0.0
efflux	vgaA	GACGGGTATTGTGGAAAGCAA	TTTCCTGTACCATTAGATCCGATAATT	AY265	0.0	0.0
efflux	vgaB	TAAAAGAGAATAAGGCGCAAGGA	TGTTTAGTAGCATGTTGCATTTTCC	AY227	4.0	0.0
efflux	vgaB	GAATGATTAAGCCCCCTTCAAAA	ATTCGTGTTTCCAACGATTTCG	AY264	2.0	0.0
deactivate	vgbA	ATACGAGCTGCCTAATAAAGGATCTT	TGTGAACCACAGGGCATTATCA	AY269	0.0	0.0
deactivate	vgbB	CAGCCGGATTCTGGTCCTT	TACGATCTCCATTCAATTGGGTAAA	AY268	0.0	0.0
**Sulphonamides (SUL)**						
protection	folA	CGAGCAGTTCCTGCCAAAG	CCCAGTCATCCGGTTCATAATC	AY208	4.0	0.0
protection	folP	CAGGCTCGTAAATTGATAGCAGAAG	CTTTCCTTGCGAATCGCTTT	AY280	10.0	3.3
protection	folP	GCGATTCGCAAGGAAAGTGA	CACATGGGCCATTTTTTCATC	AY281	10.0	0.0
protection	folP	CACGGCTTCGGCTCATGT	TGCCATCCTGTGACTAGCTACGT	AY282	6.0	0.0
protection	sul1[a]	CGCACCGGAAACATCGCTGCAC	TGAAGTTCCGCCGCAAGGCTCG	AY110	90.0	70.7
protection	sul1	CAGCGCTATGCGCTCAAG	ATCCCGCTGCGCTGAGT	AY178	0.0	0.0
protection	sul1	GCCGATGAGATCAGACGTATTG	CGCATAGCGCTGGGTTTC	AY363	100.0	100.0
protection	sul2	TCATCTGCCAAACTCGTCGTTA	GTCAAAGAACGCCGCAATGT	AY133	96.0	95.7
protection	sul2	TCCGATGGAGGCCGGTATCTGG	CGGGAATGCCATCTGCCTTGAG	AY332	94.0	89.1
protection	sul3	TCCGTTCAGCGAATTGGTGCAG	TTCGTTCACGCCTTACACCAGC	AY333	28.0	3.3
**Tetracycline (TET)**						
protection	tet(32)	CCATTACTTCGGACAACGGTAGA	CAATCTCTGTGAGGGCATTTAACA	AY54	96.0	69.6
deactivate	tet34	CTTAGCGCAAACAGCAATCAGT	CGGTGATACAGCGCGTAAACT	AY321	0.0	0.0
efflux	tet35	ACCCCATGACGTACCTGTAGAGA	CAACCCACACTGGCTACCAGTT	AY320	0.0	0.0
protection	tet(36)	AGAATACTCAGCAGAGGTCAGTTCCT	TGGTAGGTCGATAACCCGAAAAT	AY22	46.0	2.2
protection	tet36	TGCAGGAAAGACCTCCATTACAG	CTTTGTCCACACTTCCACGTACTATG	AY301	68.0	4.3
deactivate	tet37	GAGAACGTTGAAAAGGTGGTGAA	AACCAAGCCTGGATCAGTCTCA	AY302	0.0	0.0
efflux	tet38	TTAATGTGGCGGTATCTGTAGGTATT	TTGCCTGGGAAATTTAATGCTTT	AY271	0.0	0.0
efflux	tetA	GCTGTTTGTTCTGCCGGAAA	GGTTAAGTTCCTTGAACGCAAACT	AY13	0.0	0.0
efflux	tetA	CTCACCAGCCTGACCTCGAT	CACGTTGTTATAGAAGCCGCATAG	AY180	98.0	62.0

efflux	tetA/B	AGTGCGCTTTGGATGCTGTA	AGCCCCAGTAGCTCCTGTGA	AY181	90.0	5.4
efflux	tetA/B	GCCCAGTGCTGTTGTTGTCAT	TGAAAGCAAACGGCCTAAATACA	AY190	94.0	8.7
efflux	tetA(P)	AGTTGCAGATGTGTATAGTCGTAAACTATCTATT	TGCTACAAGTACGAAAACAAAACTAGAA	AY55	62.0	23.9
efflux	tetC	CATATCGCAATACATGCGAAAAA	AAAGCCGCGGTAAATAGCAA	AY182	90.0	4.3
efflux	tetC	ACTGGTAAGGTAAACGCCATTGTC	ATGCATAAACCAGCCATTGAGTAAG	AY199	92.0	3.3
efflux	tetC	TGCGTTGATGCAATTTCTATGC	GGAATGGTGCATGCAAGGAG	AY330	98.0	81.5
efflux	tetD	TGCCGCGTTTGATTACACA	CACCAGTGATCCCGGAGATAA	AY92	20.0	0.0
efflux	tetD	TGTCATCGCGCTGGTGATT	CATCCGCTTCCGGGAGAT	AY188	4.0	0.0
efflux	tetD	CTGGACGCGATGGGAATT	TCCGCTTCCGGGAGATATT	AY349	24.0	0.0
efflux	tetE	TTGGCGCTGTATGCAATGAT	CGACGACCTATGCGATCTGA	AY291	96.0	67.4
efflux	tetG	TCAACCATTGCCGATTCGA	TGGCCCGGCAATCATG	AY183	88.0	64.1
efflux	tetG	CATCAGCGCCGGTCTTATG	CCCCATGTAGCCGAACCA	AY189	92.0	89.1
efflux	tetH	TTTGGGTCATCTTACCAGCATTAA	TTGCGCATTATCATCGACAGA	AY187	64.0	2.2
efflux	tetJ	GGGTGCCGCATTAGATTACCT	TCGTCCAATGTAGAGCATCCATA	AY240	0.0	0.0
efflux	tetK	CAGCAGTCATTGGAAAATTATCTGATTATA	CCTTGTACTAACCTACCAAAAATCAAAATA	AY184	0.0	0.0
efflux	tetL	AGCCCGATTTATTCAAGGAATTG	CAAATGCTTTCCCCCTGTTCT	AY194	0.0	0.0
efflux	tetL	ATGGTTGTAGTTGCGCGCTATAT	ATCGCTGGACCGACTCCTT	AY195	54.0	3.3
protection	tetM	CATCATAGACACGCCAGGACATAT	CGCCATCTTTTGCAGAAATCA	AY186	100.0	78.3
protection	tetM	TAATATTGGAGTTTTAGCTCATGTTGATG	CCTCTCTGACGTTCTAAAAGCGTATTAT	AY193	96.0	65.2
protection	tetM	GCAATTCTACTGATTTCTGC	CTGTTTGATTACAATTTCCGC	AY329	98.0	43.5
protection	tetM[a]	GCAATTCTACTGATTTCTGC	CTGTTTGATTACAATTTCCGC	AY357	58.0	31.5
protection	tetO	ATGTGGATACTACAACGCATGAGATT	TGCCTCCACATGATATTTTTCCT	AY179	98.0	83.7
protection	tetO	CAACATTAACGGAAAGTTTATTGTATACCA	TTGACGCTCCAAATTCATTGTATC	AY192	98.0	79.3
protection	tetPB	ACACCTGGACACGCTGATTTT	ACCGTCTAGAACGCGGAATG	AY292	0.0	1.1
protection	tetPB	TGATACACCTGGACACGCTGAT	CGTCCAAAACGCGGAATG	AY293	0.0	0.0
protection	tetPB	TGGGCGACAGTAGGCTTAGAA	TGACCCTACTGAAACATTAGAAATATACCT	AY294	46.0	19.6
protection	tetPB	AGTGGTGCAAATACTGAAAAAGTTGT	TTTGTTCCTTCGTTTTGGACAGA	AY295	0.0	0.0
protection	tetPB	CTGAAGTGGAGCGATCATTCC	CCCTCAACGGCAGAAATAACTAA	AY296	0.0	0.0
protection	tetQ	CGCCTCAGAAGTAAGTTCATACACTAAG	TCGTTCATGCGGATATTATCAGAAT	AY185	100.0	89.1
regulator	tetR	ATGAGTTCGGCCAGAATTTCC	GGTTGTGCGCGAAATGATT	AY135	0.0	0.0

regulator	tetR	CGCGATAGACGCCTTCGA	TCCTGACAACGAGCCTCCTT	AY197	98.0	50.0
regulator	tetR	CGCGATGGAGCAAAAGTACAT	AGTGAAAAACCTTGTTGGCATAAAA	AY198	94.0	5.4
regulator	tetR	CAATCCATCGACAATCAC	GACAATCAGCTACTTCAC	AY381	90.0	37.0
protection	tetS	TTAAGGACAAACTTTCTGACGACATC	TGTCTCCCATTGTTCTGGTTCA	AY200	58.0	0.0
protection	tetT	CCATATAGAGGTTCCACCAAATCC	TGACCCTATTGGTAGTGGTTCTATTG	AY297	42.0	3.3
unknown	tetU	GTGGCAAAGCAACGGATTG	TGCGGGCTTGCAAAACTATC	AY69	6.0	0.0
unknown	tetU	AACAGCGGGTTAAGTGTGCAA	ATGGTATCATTCAGTTTTCCGACAAT	AY70	0.0	0.0
efflux	tetV	GCGGGAACGACGATGTATATC	CCGCTATCTCACGACCATGAT	AY228	0.0	0.0
protection	tetW	ATGAACATTCCCACCGTTATCTTT	ATATCGGCGGAGAGCTTATCC	AY191	98.0	91.3
deactivate	tetX	AAATTTGTTACCGACACGGAAGTT	CATAGCTGAAAAAATCCAGGACAGTT	AY196	98.0	88.0
**Vancomycin (VAN)**						
protection	vanA	AAAAGGCTCTGAAAACGCAGTTAT	CGGCCGTTATCTTGTAAAAACAT	AY210	2.0	0.0
protection	vanA[a]	TCTGCAATAGAGATAGCCGC	GGAGTAGCTATCCCAGCATT	AY368	2.0	0.0
protection	vanA3FP[a]	CTGTGAGGTCGGTTGTGCG	TTTGGTCCACCTCGCCA	AY4	12.0	27.2
protection	vanB	TTGTCGGCGAAGTGGATCA	AGCCTTTTTCCGGCTCGTT	AY211	0.0	0.0
protection	vanB	CCGGTCGAGGAACGAAATC	TCCTCCTGCAAAAAAAGATCAAC	AY212	8.0	0.0
protection	vanB	GGCTGTTTCGGGCTGTGA	AACAACTAACGCGGCACTGTT	AY354	2.0	0.0
protection	vanC	ACAGGGATTGGCTATGAACCAT	TGACTGGCGATGATTTGACTATG	AY66	10.0	0.0
protection	vanC	CCTGCCACAATCGATCGTT	CGGCTTCATTCGGCTTGATA	AY71	0.0	0.0
protection	vanC	AAATCAATACTATGCCGGGCTTT	CCGACCGCTGCCATCA	AY72	48.0	34.8
protection	vanC	AGGCGATAGCGGGTATTGAA	CAATCGTCAATTGCTCATTTCC	AY303	0.0	0.0
protection	vanC2/vanC3	TTTGACTGTCGGTGCTTGTGA	TCAATCGTTTCAGGCAATGG	AY304	28.0	0.0
protection	vanD	CAGAGGAACATAATGTTTCGATAAAATCT	GCCGGATTTTGTGATTCCAA	AY213	10.0	0.0
protection	vanG	ATTTGAATTGGCAGGTATACAGGTTA	TGATTTGTCTTTGTCCATACATAATGC	AY305	0.0	0.0
protection	vanHB	GAGGTTTCCGAGGCGACAA	CTCTCGGCGGCAGTCGTAT	AY215	4.0	3.3
protection	vanHD	GTGGCCGATTATACCGTCATG	CGCAGGTCATTCAGGCAAT	AY214	6.0	0.0
protection	vanRA	CCCTTACTCCCACCGAGTTTT	TTCGTCGCCCCATATCTCAT	AY216	2.0	0.0
protection	vanRA	CCACTCCGGCCTTGTCATT	GCTAACCACATTCCCCTTGTTTT	AY217	2.0	0.0
protection	vanRB	GCCCTGTCGGATGACGAA	TTACATAGTCGTCTGCCTCTGCAT	AY306	0.0	0.0
protection	vanRC	TGCGGGAAAAACTGAACGA	CCCCCCATACGGTTTTGATTA	AY307	0.0	0.0

protection	vanRC4	AGTGCTTTGGCTTATCTCGAAAA	TCCGGCAGCATCACATCTAA	AY308	20.0	0.0
protection	vanRD	TTATAATGGCAAGGATGCACTAAAGT	CGTCTACATCCGGAAGCATGA	AY309	4.0	0.0
protection	vanSA	CGCGTCATGCTTTCAAAATTC	TCCGCAGAAAGCTCAATTTGTT	AY218	2.0	0.0
protection	vanSB	GCGCGGCAAATGACAAC	TTTGCCATTTTATTCGCACTGT	AY310	8.0	3.3
protection	vanSC	ATCAACTGCGGGAGAAAAGTCT	TCCGCTGTTCCGCTTCTT	AY311	0.0	0.0
protection	vanSC	GCCATCAGCGAGTCTGATGA	CAGCTGGGATCGTTTTTCCTT	AY312	2.0	0.0
protection	vanSE	TGGCCGAAGAAGCAGGAA	CAATAATACTCGTCAAAGGAGTTCTCA	AY313	0.0	0.0
protection	vanTC	CACACGCATTTTTTCCCATCTAG	CAGCCAACAGATCATCAAAACAA	AY161	18.0	0.0
protection	vanTC	GTGGTGCCAAGGAAGTTGCT	CGTAGCCACCGCAAAAAAAT	AY314	0.0	0.0
protection	vanTC	ACAGTTGCCGCTGGTGAAG	CGTGGCTGGTCGATCAAAA	AY315	4.0	0.0
protection	vanTG	CGTGTAGCCGTTCCGTTCTT	CGGCATTACAGGTATATCTGGAAA	AY316	40.0	0.0
protection	vanWB	CGGACAAAGATACCCCCTATAAAG	AAATAGTAAATTGCTCATCTGGCACAT	AY220	8.0	0.0
protection	vanWG	ACATTTTCATTTTGGCAGCTTGTAC	CCGCCATAAGAGCCTACAATCT	AY219	54.0	0.0
protection	vanXA	CGCTAAATATGCCACTTGGGATA	TCAAAAGCGATTCAGCCAACT	AY221	2.0	0.0
protection	vanXB	AGGCACAAAATCGAAGATGCTT	GGGTATGGCTCATCAATCAACTT	AY223	16.0	0.0
protection	vanXD	TAAACCGTGTTATGGGAACGAA	GCGATAGCCGTCCCATAAGA	AY222	4.0	0.0
protection	vanYB	GGCTAAAGCGGAAGCAGAAA	GATATCCACAGCAAGACCAAGCT	AY317	4.0	0.0
protection	vanYD	AAGGCGATACCCTGACTGTCA	ATTGCCGGACGGAAGCA	AY318	10.0	0.0
protection	vanYD	CAAACGGAAGAGAGGTCACTTACA	CGGACGGTAATAGGGACTGTTC	AY319	8.0	0.0
**Other**						
protection	mcr-1[a]	ACACTTATGGCACGGTCTATG	GCACACCCAAACCAATGATAC	AY80	0.0	0.0
deactivate	dfrA1	GGAATGGCCCTGATATTCCA	AGTCTTGCGTCCAACCAACAG	AY58	96.0	48.9
deactivate	dfrA1	TTCAGGTGGTGGGGAGATATAC	TTAGAGGCGAAGTCTTGGGTAA	AY334	0.0	0.0
deactivate	dfrA12	CCTCTACCGAACCGTCACACA	GCGACAGCGTTGAAACAACTAC	AY59	86.0	12.0
deactivate	merA	GTGCCGTCCAAGATCATG	GGTGGAAGTCCAGTAGGGTGA	AY331	82.0	82.6
deactivate	bacA	CGGCTTCGTGACCTCGTT	ACAATGCGATACCAGGCAAAT	AY157	10.0	1.1
deactivate	bacA	TTCCACGACACGATTAAGTCATTG	CGGCTCTTTCGGCTTCAG	AY158	96.0	34.8
protection	fabK	TTTCAGCTCAGCACTTTGGTCAT	AAGGCATCTTTTTCAGCCAGTTC	AY279	0.0	3.3
deactivate	fosB	TCACTGTAACTAATGAAGCATTAGACCAT	CCATCTGGATCTGTAAAGTAAAGAGATC	AY143	0.0	0.0
deactivate	fosX	GATTAAGCCATATCACTTTAATTGTGAAAG	TCTCCTTCCATAATGCAAATCCA	AY76	0.0	0.0

regulator	imiR	CCGGACTAGAGCTTCATGTAAGC	CCCACGCGGTACTCTTGTAAA	AY65	0.0	0.0
deactivate	nimE	TGCGCCAAGATAGGGCATA	GTCGTGAATTCGGCAGGTTTA	AY152	50.0	0.0
deactivate	nisB	GGGAGAGTTGCCGATGTTGTA	AGCCACTCGTTAAAGGGCAAT	AY79	6.0	0.0
deactivate	nisB	CGGTTGAACGGCGTGAA	TTTCCACCCAGGTTTGCTACTATT	AY348	14.0	0.0
deactivate	yyaR	CCGTTGCAAGAAGATTATAGAAAAAA	CAAGCATAAGACCGCATAAATGAT	AY16	0.0	0.0
**Integrase**						
MGE	intI1[a]	GATCGGTCGAATGCGTGT	GCCTTGATGTTACCCGAGAG	AY45	100.0	97.8
MGE	intI1	CGAAGTCGAGGCATTTCTGTC	GCCTTCCAGAAAACCGAGGA	AY336	100.0	97.8
MGE	intI1	GCCTTGATGTTACCCGAGAG	GATCGGTCGAATGCGTGT	AY337	96.0	95.7
MGE	intI1	CGAACGAGTGGCGGAGGGTG	TACCCGAGAGCTTGGCACCCA	AY359	96.0	94.6
MGE	intI1	CTGGATTTCGATCACGGCACG	ACATGCGTGTAAATCATCGTCG	AY364	86.0	84.8
MGE	intI2	TGCTTTTCCCACCCTTACC	GACGGCTACCCTCTGTTATCTC	AY338	96.0	15.2
MGE	intI3	GCCACCACTTGTTTGAGGA	GGATGTCTGTGCCTGCTTG	AY339	94.0	66.3
**Transposase**						
MGE	IS613	AGGTTCGGACTCAATGCAACA	TTCAGCACATACCGCCTTGAT	AY26	68.0	75.0
MGE	tnpA	GCCGCACTGTCGATTTTTATC	GCGGGATCTGCCACTTCTT	AY201	100.0	92.4
MGE	tnpA	CCGATCACGGAAAGCTCAAG	GGCTCGCATGACTTCGAATC	AY202	98.0	95.7
MGE	tnpA	GGGCGGGTCGATTGAAA	GTGGGCGGGATCTGCTT	AY203	92.0	90.2
MGE	tnpA	CATCATCGGACGGACAGAATT	GTCGGAGATGTGGGTGTAGAAAGT	AY204	90.0	78.3
MGE	tnpA	GAAACCGATGCTACAATATCCAATTT	CAGCACCGTTTGCAGTGTAAG	AY205	100.0	91.3
MGE	tnpA	TGCAGATGGTTTAACCTTGGATATTT	TCGGTTCATCAAACTGCTTCAC	AY206	96.0	66.3
MGE	tnpA	AATTGATGCGGACGGCTTAA	TCACCAAACTGTTTATGGAGTCGTT	AY207	96.0	81.5
MGE	Tp614	GGAAATCAACGGCATCCAGTT	CATCCATGCGCTTTTGTCTCT	AY25	100.0	80.4
MGE	trfA	ACGAAGAAATGGTTGTCCTGTTC	CGTCAGCTTGCGGTACTTCTC	AY358	78.0	66.3
**Insertional sequence**						
MGE	IS1111	GTCTTAAGGTGGGCTGCGTG	CCCCGAATCTCATTGATCAGC	AY376	0.0	1.1
MGE	IS1133	GCAGCGTCGGGTTGGA	ACGCGTTCGAACAACTGTAATG	AY383	82.0	2.2
MGE	ISAba3	TCAGAGGCAGCGGTATACGA	GGTTGATTCAGTTAAAGTACGTAAAACTTT	AY371	100.0	91.3
MGE	ISEfm1	AGGTGTCCATGACGTGAAAGTG	TCCTTTGTCCCCTAGGATATTGG	AY372	92.0	5.4
MGE	ISPps	CACACTGCAAAAACGCATCCT	TGTCTTTGGCGTCACAGTTCTC	AY369	100.0	95.7

MGE	ISSm2	TGGATCGACCGGTTCCAT	GCTGACCGAGCTGTCCATGT	AY370	100.0	100.0
MGE	orf37-IS26	GCCGGGTTGTGCAAATAGAC	TGGCAATCTGTCGCTGCTG	AY365	50.0	0.0
MGE	orf39-IS26	GCGCGTCGAGCATCAATAG	CAGTTGTGCTGCTGGTGGTC	AY366	82.0	5.4
MGE	Tn5	CAGCATAAAAAATCCCGACAACA	CCCCGCAACAGACATACGT	AY384	48.0	0.0
**Plasmid-associated**						
MGE	IncN_rep	AGTTCACCACCTACTCGCTCCG	CAAGTTCTTCTGTTGGGATTCCG	AY340	n.a.	n.a.
MGE	IncN_oriT	TTGGGCTTCATAGTACCC	GTGTGATAGCGTGATTTATGC	AY341	n.a.	n.a.
MGE	IncP_oriT	CAGCCTCGCAGAGCAGGAT	CAGCCGGGCAGGATAGGTGAAGT	AY342	n.a.	n.a.
MGE	IncQ_oriT	TTCGCGCTCGTTGTTCTTCGAGC	GCCGTTAGGCCAGTTTCTCG	AY343	n.a.	n.a.
MGE	IncW_trwAB	AGCGTATGAAGCCCGTGAAGGG	AAAGATAAGCGGCAGGACAATAACG	AY344	n.a.	n.a.
MGE	PAMBL	CAGGCTCTTAATGTGATA	TTATGCTCAATACTCGTG	AY377	n.a.	n.a.
MGE	pAKD1	GGTAAGATTACCGATAAACT	GTTCGTGAAGAAGATGTA	AY380	n.a.	n.a.
MGE	pNI105	CGCTAAGGATGTTTACAC	CTCAACCGTTCTAGGATT	AY379	n.a.	n.a.
MGE	repA	CCCCCAGGACTTGCGAGCG	GAGGCATGCACGCCGACCA	AY378	n.a.	n.a.
	**Gene identification**	**Forward Primer**	**Reverse Primer**	**Assay**	**% Positive Influent**	**% Positive Effluent**
**Housekeeping genes (HK)**						
	16S rRNA	GGGTTGCGCTCGTTGC	ATGGYTGTCGTCAGCTCGTG	AY1	100.0	100.0
	16S rRNA (2)[a]	CCTACGGGAGGCAGCAG	ATTACCGCGGCTGCTGGC	AY2	100.0	100.0
	gapA	CCGTTGAAGTGAAAGACGGTC	AACCACTTTCTTCGCACCAGC	AY347	96.0	31.5
	mdh	AAGAAACGGGCGTACTGACC	GTGGCTGATCTGACCAAACG	AY346	94.0	34.8
	rpoB	CGAACATCGGTCTGATCAACTC	GTTGCATGTTCGCACCCAT	AY345	44.0	18.5
	uidA	AACCACGCGTCTGTTGACTG	CCCGGTTGCCAGAGGTG	AY75	78.0	19.6
**Aminoglycosides (AMG)**						
deactivate	aac3-VI	CGTCACTTATTCGATGCCCTTAC	GTCGGGCGCGGCATA	AY93	34.0	23.9
deactivate	aac(6)I1	GACCGGATTAAGGCCGATG	CTTGCCTTGATATTCAGTTTTTATAACCA	AY5	0.0	0.0
deactivate	aac(6)-II	CGACCCGACTCCGAACAA	GCACGAATCCTGCCTTCTCA	AY8	84.0	20.7
deactivate	aac(6)-Ib	GTTTGAGAGGCAAGGTACCGTAA	GAATGCCTGGCGTGTTTGA	AY94	96.0	70.7
deactivate	aac(6)-Ib	CGTCGCCGAGCAACTTG	CGGTACCTTGCCTCTCAAACC	AY95	96.0	64.1
deactivate	aac(6)-Ib	AGAAGCACGCCCGACACTT	GCTCTCCATTCAGCATTGCA	AY172	98.0	67.4

deactivate	aac(6)-Iy	GCTTTGCGGATGCCTCAAT	GGAGAACAAAAATACCTTCAAGGAAA	AY7	0.0	0.0
deactivate	aadA	AGCTAAGCGCGAACTGCAAT	TGGCTCGAAGATACCTGCAA	AY96	100.0	98.9
deactivate	aadA	ACGGCTCCGCAGTGGAT	GGCCACAGTAACCAACAAATCA	AY97	100.0	87.0
deactivate	aadA	GTTGTGCACGACGACATCATT	GGCTCGAAGATACCTGCAAGAA	AY99	100.0	97.8
deactivate	aadA	CTTGTCGTGCATGACGACATC	TCGAAGATACCCGCAAGAATG	AY101	98.0	88.0
deactivate	aadA	CAATGACATTCTTGCGGGTATC	GACCTACCAAGGCAACGCTATG	AY167	100.0	98.9
deactivate	aadA	CGAGATTCTCCGCGCTGTA	GCTGCCATTCTCCAAATTGC	AY169	100.0	96.7
deactivate	aadA5	ATCACGATCTTGCGATTTTGCT	CTGCGGATGGGCCTAGAAG	AY98	96.0	67.4
deactivate	aadA5	GTTCTTGCTCTTGCTCGCATT	GATGCTCGGCAGGCAAAC	AY100	98.0	71.7
deactivate	aadA9	CGCGGCAAGCCTATCTTG	CAAATCAGCGACCGCAGACT	AY168	0.0	0.0
deactivate	aadA9	GGATGCACGCTTGGATGAA	CCTCTAGCGGCCGGAGTATT	AY173	0.0	0.0
deactivate	aadD	CCGACAACATTTCTACCATCCTT	ACCGAAGCGCTCGTCGTATA	AY151	52.0	8.7
deactivate	aadE	TACCTTATTGCCCTTGGAAGAGTTA	GGAACTATGTCCCTTTTAATTCTACAATCT	AY174	96.0	28.3
deactivate	acc	CCCTGCGTTGTGGCTATGT	TTGGCCACGCCAATCC	AY382	0.0	0.0
deactivate	aph	TTTCAGCAAGTGGATCATGTTAAAAT	CCAAGCTGTTTCCACTGTTTTTC	AY166	0.0	0.0
deactivate	aphA1/7	TGAACAAGTCTGGAAAGAAATGCA	CCTATTAATTTCCCCTCGTCAAAAA	AY170	96.0	38.0
deactivate	aphA3	AAAAGCCCGAAGAGGAACTTG	CATCTTTCACAAAGATGTTGCTGTCT	AY14	96.0	18.5
deactivate	aphA3	CGGAATTGAAAAAACTGATCGAA	ATACCGGCTGTCCGTCATTT	AY171	96.0	35.9
deactivate	aph(2)-Ib	TGAGCAGTATCATAAGTTGAGTGAAAAG	GACAGAACAATCAATCTCTATGGAATG	AY104	60.0	0.0
deactivate	aph(2)-Id	TAAGGATATACCGACAGTTTTGGAAA	TTTAATCCCTCTTCATACCAATCCATA	AY105	6.0	0.0
deactivate	aph6	CCCATCCCATGTGTAAGGAAA	GCCACCGCTTCTGCTGTAC	AY15	0.0	0.0
deactivate	sat4	GAATGGGCAAAGCATAAAAACTTG	CCGATTTTGAAACCACAATTATGATA	AY49	96.0	16.3
deactivate	spcN	AAAAGTTCGATGAAACACGCCTAT	TCCAGTGGTAGTCCCCGAATC	AY286	0.0	0.0
deactivate	spcN	CAGAATCTTCCTGAAAAGTTTGATGAA	CGCAGACACGCCGAATC	AY287	0.0	0.0
deactivate	str	AATGAGTTTTGGAGTGTCTCAACGTA	AATCAAAACCCCTATTAAAGCCAAT	AY175	90.0	2.2
deactivate	strA	CCGGTGGCATTTGAGAAAAA	GTGGCTCAACCTGCGAAAAG	AY176	90.0	10.9
deactivate	strB	GCTCGGTCGTGAGAACAATCT	CAATTTCGGTCGCCTGGTAGT	AY177	100.0	95.7
**Amphenicols (AMP)**						
deactivate	catA1	GGGTGAGTTTCACCAGTTTTGATT	CACCTTGTCGCCTTGCGTATA	AY130	94.0	4.3
deactivate	catB3	GCACTCGATGCCTTCCAAAA	AGAGCCGATCCAAACGTCAT	AY51	96.0	41.3

deactivate	catB8	CACTCGACGCCTTCCAAAG	CCGAGCCTATCCAGACATCATT	AY52	86.0	17.4
efflux	ceoA	ATCAACACGGACCAGGACAAG	GGAAAGTCCGCTCACGATGA	AY53	0.0	0.0
efflux	cmlA	AATTTTGCCGATTATTGCTGAAA	GATTGTCATCATTCGTTTATCACCAA	AY20	0.0	0.0
efflux	cmlA	TAGGAAGCATCGGAACGTTGAT	CAGACCGAGCACGACTGTTG	AY127	96.0	43.5
efflux	cmlA	AGGAAGCATCGGAACGTTGA	ACAGACCGAGCACGACTGTTG	AY128	56.0	40.2
efflux	cmlA	GCGCTCTTCGAGGATTCG	CCGCCCAAGCAGAAGTAGAC	AY375	96.0	43.5
efflux	cmxA	GCGATCGCCATCCTCTGT	TCGACACGGAGCCTTGGT	AY129	86.0	35.9
efflux	floR	ATTGTCTTCACGGTGTCCGTTA	CCGCGATGTCGTCGAACT	AY56	92.0	44.6
efflux	floR	TCGTCATCTACGGCCTTTTC	CTTGACTTGATCCAGAGGGC	AY335	8.0	0.0
efflux	mdtL	TGCTGATCGGGATTCTGATTG	CAGGCGCGACGAACATAAT	AY88	96.0	33.7
efflux	rarD	GCGGGTGTGGTCACTACGAT	AGCGTTGGGCCGATATACTG	AY131	28.0	1.1
efflux	rarD	TGACGCATCGCGTGATCT	AAATTTTCTGTGGCGTCTGAATC	AY132	96.0	29.3
efflux	yidY/mdtL	GCAGTTGCATATCGCCTTCTC	CTTCCCGGCAAACAGCAT	AY87	24.0	5.4
**Beta-lactams (BL)**						
deactivate	ampC	GCAGCACGCCCCGTAA	TGTACCCATGATGCGCGTACT	AY30	86.0	23.9
deactivate	ampC	AACAAAAGATCCCCGGTATGG	ACGCCCGTAAATGTTTTGCT	AY31	96.0	43.5
deactivate	ampC	TCCGGTGACGCGACAGA	CAGCACGCCGGTGAAAGT	AY33	2.0	0.0
deactivate	ampC	CCGCCCAGAGCAAGGACTA	GCTCGACTTCACGCCGTAAG	AY114	0.0	0.0
deactivate	ampC	GCAGCGAAGCGTCAGTCA	AGATCCGTGGCCGCATAA	AY258	4.0	0.0
deactivate	bla1	GCAAGTTGAAGCGAAAGAAAAGA	TACCAGTATCAATCGCATATACACCTAA	AY39	0.0	0.0
deactivate	blaACC	CACACAGCTGATGGCTTATCTAAAA	AATAAACGCGATGGGTTCCA	AY28	0.0	0.0
deactivate	blaACT	TGGCGTATCGGGTCAATGT	CTCCACGGGCCAGTTGAG	AY29	12.0	0.0
deactivate	blaACT	CTGTTCGAGCTGGGTTCTATAAGTAAA	CAGTATCTGGTCACCGGATCGT	AY109	46.0	0.0
deactivate	blaCMY	CCGCGGCGAAATTAAGC	GCCACTGTTTGCCTGTCAGTT	AY32	88.0	12.0
deactivate	blaCMY	AAAGCCTCATGGGTGCATAAA	ATAGCTTTTGTTTGCCAGCATCA	AY108	94.0	16.3
deactivate	blaCMY	GCGAGCAGCCTGAAGCA	CGGATGGGCTTGTCCTCTT	AY111	84.0	20.7
deactivate	blaCTX-M	GGAGGCGTGACGGCTTTT	TTCAGTGCGATCCAGACGAA	AY116	34.0	0.0
deactivate	blaCTX-M	GCCGCGGTGCTGAAGA	ATCGGATTATAGTTAACCAGGTCAGATTT	AY117	74.0	8.7
deactivate	blaCTX-M	CGATACCACCACGCCGTTA	GCATTGCCCAACGTCAGATT	AY118	8.0	0.0
deactivate	blaCTX-M	CTTGGCGTTGCGCTGAT	CGTTCATCGGCACGGTAGA	AY119	18.0	6.5

deactivate	blaCTX-M	GCGATAACGTGGCGATGAAT	GTCGAGACGGAACGTTTCGT	AY162	76.0	3.3
deactivate	blaCTX-M	CACAGTTGGTGACGTGGCTTAA	CTCCGCTGCCGGTTTTATC	AY165	38.0	3.3
deactivate	blaCTX-M	CGTCACGCTGTTGTTAGGAA	CGCTCATCAGCACGATAAAG	AY324	84.0	7.6
deactivate	blaCTX-M[a]	CGATGTGCAGTACCAGTAA	GCAATGGGATTGTAGTTAA	AY326	16.0	1.1
deactivate	blaCTX-M[a]	CTATGGCACCACCAACGATA	ACGGCTTTCTGCCTTAGGTT	AY360	84.0	7.6
deactivate	blaDHA	TGGCCGCAGCAGAAAGA	CCGTTTTATGCACCCAGGAA	AY112	14.0	0.0
deactivate	blaFOX	GGTTTGCCGCTGCAGTTC	GCGGCCAGGTGACCAA	AY113	38.0	45.7
deactivate	blaGES	GCAATGTGCTCAACGTTCAAG	GTGCCTGAGTCAATTCTTTCAAAG	AY120	100.0	56.5
deactivate	blaIMP	AACACGGTTTGGTGGTTCTTGTA	GCGCTCCACAAACCAATTG	AY148	12.0	19.6
deactivate	blaIMP	AAGGCAGCATTTCCTCTCATTTT	GGATAGATCGAGAATTAAGCCACTCT	AY242	8.0	46.7
deactivate	blaIMP	GGAATAGAGTGGCTTAATTC	GGTTTAACAAAACAACCACC	AY327	6.0	23.9
deactivate	blaKPC	CAGCTCATTCAAGGGCTTTC	GGCGGCGTTATCACTGTATT	AY323	14.0	0.0
deactivate	blaKPC	GCCGCCGTGCAATACAGT	GCCGCCCAACTCCTTCA	AY367	16.0	2.2
deactivate	blaL1	CACCGGGTTACCAGCTGAAG	GCGAAGCTGCGCTTGTAGTC	AY48	0.0	1.1
deactivate	blaMOX/blaCMY	CTATGTCAATGTGCCGAAGCA	GGCTTGTCCTCTTTCGAATAGC	AY34	94.0	21.7
deactivate	blaNDM	GGCCACACCAGTGACAATATCA	CAGGCAGCCACCAAAAGC	AY362	0.0	0.0
deactivate	blaOCH	GGCGACTTGCGCCGTAT	TTTTCTGCTCGGCCATGAG	AY35	2.0	0.0
deactivate	blaOKP	GCCGCCATCACCATGAG	GGTGACGTTGTCACCGATCTG	AY40	6.0	0.0
deactivate	blaOXA	CGCAATTATCGGCCTAGAAACT	TTGGCTTTCCGTCCCATTT	AY44	100.0	90.2
deactivate	blaOXA	CGGATGGTTTGAAGGGTTTATTAT	TCTTGGCTTTTATGCTTGATGTTAA	AY115	96.0	83.7
deactivate	blaOXA	GCAATTGCCTTTTAAACCTGA	CTGCCTTTTCAACAAAACCC	AY322	96.0	35.9
deactivate	blaOXA[a]	CACTTACAGGAAACTTGGGGTCG	AGTGTGTTTAGAATGGTGATC	AY361	96.0	78.3
deactivate	blaOXY	CGTTCAGGCGGCAGGTT	GCCGCGATATAAGATTTGAGAATT	AY42	18.0	0.0
deactivate	blaPDC	CGCCGTACAACCGGTGAT	GAAGTAATGCGGTTCTCCTTTCA	AY36	2.0	0.0
deactivate	blaPER	TGCTGGTTGCTGTTTTTGTGA	CCTGCGCAATGATAGCTTCAT	AY241	80.0	9.8
deactivate	blaPSE	TTGTGACCTATTCCCCTGTAATAGAA	TGCGAAGCACGCATCATC	AY43	70.0	10.9
deactivate	blaROB	GCAAAGGCATGACGATTGC	CGCGCTGTTGTCGCTAAA	AY41	10.0	1.1
deactivate	blaSFO	CCGCCGCCATCCAGTA	GGGCCGCCAAGATGCT	AY121	64.0	6.5
deactivate	blaSHV	TCCCATGATGAGCACCTTTAAA	TTCGTCACCGGCATCCA	AY37	96.0	6.5
deactivate	blaSHV	CTTTCCCATGATGAGCACCTTT	TCCTGCTGGCGATAGTGGAT	AY163	90.0	4.3

deactivate	blaSHV[a]	CGCTTTCCCATGATGAGCACCTTT	TCCTGCTGGCGATAGTGGATCTTT	AY272	58.0	3.3
deactivate	blaSHV	GCGTTATTTTCGCCTGTGTA	AGGTGCTCATCATGGGAAAG	AY325	76.0	0.0
deactivate	blaSRT	CAGCCGCTGATGAAAAAATATG	CAGCGAGCCCACTTCGA	AY259	0.0	0.0
deactivate	blaTEM[a]	TTCCTGTTTTTGCTCACCCAG	CTCAAGGATCTTACCGCTGTTG	AY3	94.0	45.7
deactivate	blaTEM	AGCATCTTACGGATGGCATGA	TCCTCCGATCGTTGTCAGAAGT	AY164	96.0	53.3
deactivate	blaTLA	ACACTTTGCCATTGCTGTTTATGT	TGCAAATTTCGGCAATAATCTTT	AY122	0.0	0.0
deactivate	blaVEB	CCCGATGCAAAGCGTTATG	GAAAGATTCCCTTTATCTATCTCAGACAA	AY38	100.0	45.7
deactivate	blaVIM	GCACTTCTCGCGGAGATTG	CGACGGTGATGCGTACGTT	AY147	36.0	0.0
deactivate	blaVIM2[a]	CCTCCATTGAGCGGATTCA	GCCGTGCCCCGGAA	AY260	10.0	0.0
deactivate	blaZ	GGAGATAAAGTAACAAATCCAGTTAGATATGA	TGCTTAATTTTCCATTTGCGATAAG	AY123	2.0	0.0
deactivate	ccrA	GCAGCGTTGCTGGACACA	GTTCGGGATAAACGTGGTGACT	AY24	22.0	0.0
deactivate	cepA	AGTTGCGCAGAACAGTCCTCTT	TCGTATCTTGCCCGTCGATAAT	AY107	82.0	18.5
deactivate	cfxA	TCATTCCTCGTTCAAGTTTTCAGA	TGCAGCACCAAGAGGAGATGT	AY106	98.0	84.8
deactivate	cphA	GCGAGCTGCACAAGCTGAT	CGGCCCAGTCGCTCTTC	AY46	90.0	22.8
deactivate	cphA	GTGCTGATGGCGAGTTTCTG	GGTGTGGTAGTTGGTGTTGATCAC	AY47	92.0	21.7
protection	mecA	GGTTACGGACAAGGTGAAATACTGAT	TGTCTTTTAATAAGTGAGGTGCGTTAATA	AY155	0.0	0.0
protection	mecA[a]	GTTGTAGTTGTCGGGTTTGG	CTTCCACATACCATCTTCTTTAAC	AY284	0.0	3.3
protection	pbp	CCGGTGCCATTGGTTTAGA	AAAATAGCCGCCCCAAGATT	AY154	14.0	0.0
protection	pbp2x	TTTCATAAGTATCTGGACATGGAAGAA	CCAAAGGAAACTTGCTTGAGATTAG	AY237	0.0	0.0
protection	pbp5	GGCGAACTTCTAATTAATCCTATCCA	CGCCGATGACATTCTTCTTATCTT	AY153	24.0	0.0
protection	penA	AGACGGTAACGTATAACTTTTTGAAAGA	GCGTGTAGCCGGCAATG	AY236	0.0	0.0
deactivate	sdeB	GGCATGCAGAAAGTGTTTATGC	TTAAGTGCGGAAGCGGTAGTG	AY353	0.0	0.0
**Quinolones (QUI)**						
efflux	pmrA	TTTGCAGGTTTTGTTCCTAATGC	GCAGAGCCTGATTTCTCCTTTG	AY239	0.0	0.0
protection	qnrA	AGGATTTCTCACGCCAGGATT	CCGCTTTCAATGAAACTGCAA	AY248	14.0	1.1
protection	qnrB	GCGACGTTCAGTGGTTCAGA	GCTGCTCGCCAGTCGAA	AY328	58.0	5.4
protection	qnrSrtF11[a]	GACGTGCTAACTTGCGTGAT	TGGCATTGTTGGAAACTTG	AY6	96.0	56.5
**Multidrug Resistance (MDR)**						
efflux	acrA	CAACGATCGGACGGGTTTC	TGGCGATGCCACCGTACT	AY61	92.0	8.7
efflux	acrA	GGTCTATCACCCTACGCGCTATC	GCGCGCACGAACATACC	AY62	80.0	12.0

efflux	acrA	CAGACCCGCATCGCATATT	CGACAATTTCGCGCTCATG	AY249	0.0	0.0
efflux	acrA	TACTTTGCGCGCCATCTTC	CGTGCGCGAACGAACAT	AY256	28.0	2.2
efflux	acrA	CGTGCGCGAACGAACA	ACTTTGCGCGCCATCTTC	AY257	50.0	13.0
efflux	acrB	AGTCGGTGTTCGCCGTTAAC	CAAGGAAACGAACGCAATACC	AY9	96.0	23.9
efflux	acrB	TGGTAGTGGGCGTCATTAACAC	GGCAACGTAATCCGAAATATCC	AY10	0.0	0.0
efflux	acrF	GCGGCCAGGCACAAAA	TACGCTCTTCCCACGGTTTC	AY11	92.0	20.7
regulator	acrR	GCGCTGGAGACACGACAAC	GCCTTGCTGCGAGAACAAA	AY102	76.0	2.2
regulator	acrR	GATGATACCCCCTGCTGTGAGA	ACCAAACAAGAAGCGCAAGAA	AY103	96.0	34.8
regulator	acrR	TGCAACACGCGCTTTCTC	ACGATTGCGGGCAGGTT	AY350	22.0	17.4
efflux	adeA	CAGTTCGAGCGCCTATTTCTG	CGCCCTGACCGACCAAT	AY12	18.0	0.0
efflux	bexA	GCGGATCTCTGGTCAGCAA	TGATTGATGGTTCCCCGTACA	AY27	84.0	15.2
protection	cfr	GCAAAATTCAGAGCAAGTTACGAA	AAAATGACTCCCAACCTGCTTTAT	AY277	0.0	0.0
efflux	cmeA	GCAGCAAAGAAGAAGCACCAA	AGCAGGGTAAGTAAAACTAAGTGGTAAATCT	AY50	0.0	0.0
efflux	cmr	CGGCATCGTCAGTGGAATT	CGGTTCCGAAAAAGATGGAA	AY57	0.0	0.0
efflux	emrB/qacA	CTTTTCTCTAACCGTACATTATCTACGATAAA	AGAACGTAGCGACTGATAAAATGCT	AY156	0.0	0.0
efflux	emrB/qacA	GCAGTAGAAGGAACGATTGTTAGTACAG	TGCGTAAACCCAGCTAACAAGTT	AY352	0.0	0.0
efflux	emrD	CTCAGCAGTATGGTGGTAAGCATT	ACCAGGCGCCGAAGAAC	AY64	16.0	0.0
regulator	marR	GCGGCGTACTGGTGAAGCTA	TGCCCTGGTCGTTGATGA	AY254	0.0	0.0
regulator	marR	TCTGGCGTTAGCTTCACCAGTAC	GTGCAAAGGCTGGATCGAA	AY255	0.0	0.0
regulator	marR	GCTGTTGATGACATTGCTCACA	CGGCGTACTGGTGAAGCTAAC	AY355	12.0	0.0
efflux	mdsA	CGGAGTCCATCGACCATTTG	ATCGTCGGCAAGGAGAATCA	AY253	0.0	0.0
efflux	mdtA	CCTAACGGGCGTGACTTCA	TTCACCTGTTTCAAGGGTCAAA	AY78	0.0	0.0
efflux	mdtE	CGTCGGCGCACTCGTT	TCCAGACGTTGTACGGTAACCA	AY81	84.0	16.3
efflux	mdtF	CCACCATCGGGCTTTCC	CCCTTCTTTCTGCATCATCTCA	AY373	96.0	32.6
efflux	mdtG	TGGCACAAAATATCTGGCAGTT	TTGTGTGGCGATAAGAGCATTAG	AY82	96.0	31.5
efflux	mdtG	TTATCTGTTTTCTGCTCACCTTCTTTT	GCGTGGTGACAAACAGGCTTA	AY83	0.0	0.0
efflux	mdtH	CGCGTGAAACCTTAAGTGCTT	AGACGGCTAAACCCCATATAGCT	AY85	96.0	26.1
efflux	mdtH	CTGCCGTTAAATGGATGTATGC	ACTCCAGCGGGCGATAGG	AY86	94.0	33.7
efflux	mepA	ATCGGTCGCTCTTCGTTCAC	ATAAATAGGATCGAGCTGCTGGAT	AY245	8.0	5.4
efflux	mexA	AGGACAACGCTATGCAACGAA	CCGGAAAGGGCCGAAAT	AY89	4.0	0.0

efflux	mexB	CTGGAGATCGACGACGAGAAG	GAAATCGTTGACGTAGCTGGAA	AY374	6.0	0.0
efflux	mexD	TTGCCACTGGCTTTCATGAG	CACTGCGGAGAACTGTCTGTAGA	AY90	0.0	0.0
efflux	mexE	GGTCAGCACCGACAAGGTCTAC	AGCTCGACGTACTTGAGGAACAC	AY246	90.0	20.7
efflux	mexF	CCGCGAGAAGGCCAAGA	TTGAGTTCGGCGGTGATGA	AY247	46.0	37.0
efflux	mtrC	GGACGGGAAGATGGTCCAA	CGTAGCGTTCCGGTTCGAT	AY250	0.0	0.0
efflux	mtrD	TGCGCGTAGTCGTTCATCTC	CGTTCCAATTTCCTGATGATTG	AY230	0.0	0.0
efflux	mtrD	GGTCGGCACGCTCTTGTC	TGAAGAATTTGCGCACCACTAC	AY232	24.0	0.0
efflux	mtrD	CCGCCAAGCCGATATAGACA	GGCCGGGTTGCCAAA	AY233	0.0	2.2
efflux	mtrE	CGATGTGTCGTTTTGGAAGGT	CCTGCACCATGATTCCTCAATA	AY231	0.0	0.0
efflux	oprD	ATGAAGTGGAGCGCCATTG	GGCCACGGCGAACTGA	AY234	34.0	17.4
efflux	oprJ	ACGAGAGTGGCGTCGACAA	AAGGCGATCTCGTTGAGGAA	AY235	44.0	23.9
efflux	qac	GGAGATTTAGCTCATGTAGCTGAAGAA	AAGCTGTTTTATCCCCGTAGCTTTA	AY356	4.0	1.1
efflux	qacA/qacB	TGGCAATAGGAGCTATGGTGTTT	AAGGTAACACTATTTTCGGTCCAAATC	AY262	2.0	0.0
efflux	qacEdelta1	TCGCAACATCCGCATTAAAA	ATGGATTTCAGAACCAGAGAAAGAAA	AY126	100.0	97.8
efflux	qacEdelta1	CCCCTTCCGCCGTTGT	CGACCAGACTGCATAAGCAACA	AY159	100.0	98.9
efflux	qacEdelta1	GTCGGTGTTGCTTATGCAGTCT	CAACCAGGCAATGGCTGTAA	AY351	100.0	100.0
efflux	qacG	CAATAATAACCGAAATAATAGGGACAAGTT	AATAAGTGTTCCTAGTGTTGGCCATAG	AY261	0.0	0.0
efflux	qacH	GTGGCAGCTATCGCTTGGAT	CCAACGAACGCCCACAA	AY124	88.0	47.8
efflux	qacH	CATCGTGCTTGTGGCAGCTA	TGAACGCCCAGAAGTCTAGTTTT	AY125	100.0	94.6
efflux	sdeB	CACTACCGCTTCCGCACTTAA	TGAAAAAACGGGAAAAGTCCAT	AY160	0.0	0.0
efflux	tolC	GGCCGAGAACCTGATGCA	AGACTTACGCAATTCCGGGTTA	AY298	96.0	32.6
efflux	tolC	CAGGCAGAGAACCTGATGCA	CGCAATTCCGGGTTGCT	AY299	68.0	3.3
efflux	tolC	GCCAGGCAGAGAACCTGATG	CGCAATTCCGGGTTGCT	AY300	66.0	6.5
efflux	ttgA	ACGCCAATGCCAAACGATT	GTCACGGCGCAGCTTGA	AY243	50.0	9.8
efflux	ttgB	TCGCCCTGGATGTACACCTT	ACCATTGCCGACATCAACAAC	AY244	90.0	34.8
efflux	yceL/mdtH	TCGGGATGGTGGGCAAT	CGATAACCGAGCCGATGTAGA	AY84	0.0	0.0
**Macrolide-Lincosamide-Streptogramin B (MLS$_B$)**						
efflux	carB	GGAGTGAGGCTGACCGTAGAAG	ATCGGCGAAACGCACAAA	AY288	0.0	0.0
deactivate	ereA	CCTGTGGTACGGAGAATTCATGT	ACCGCATTCGCTTTGCTT	AY60	100.0	58.7
deactivate	ereB	GCTTTATTTCAGGAGGCGGAAT	TTTTAAATGCCACAGCACAGAATC	AY63	72.0	4.3

protection	erm34	GCGCGTTGACGACGATTT	TGGTCATACTCGACGGCTAGAAC	AY134	0.0	0.0
protection	erm(35)	TTGAAAACGATGTTGCATTAAGTCA	TCTATAATCACAACTAACCACTTGAACGT	AY21	28.0	3.3
protection	erm(36)	GGCGGACCGACTTGCAT	TCTGCGTTGACGACGGTTAC	AY91	0.0	0.0
protection	ermA	TTGAGAAGGGATTTGCGAAAAG	ATATCCATCTCCACCATTAATAGTAAACC	AY274	2.0	2.2
protection	ermA/ermTR	ACATTTTACCAAGGAACTTGTGGAA	GTGGCATGACATAAACCTTCATCA	AY283	2.0	0.0
protection	ermB	TAAAGGGCATTTAACGACGAAACT	TTTATACCTCTGTTTGTTAGGGAATTGAA	AY136	96.0	82.6
protection	ermC	TTTGAAATCGGCTCAGGAAAA	ATGGTCTATTTCAATGGCAGTTACG	AY275	2.0	4.3
protection	ermD	GGACTCGGCAATGGTCAGAA	CCCCGAAACGCAATATAATGTT	AY18	0.0	0.0
protection	ermF	CAGCTTTGGTTGAACATTTACGAA	AAATTCCTAAAATCACAACCGACAA	AY23	100.0	94.6
protection	ermK	GTTTGATATTGGCATTGTCAGAGAAA	ACCATTGCCGAGTCCACTTT	AY17	0.0	0.0
protection	ermK	GAGCCGCAAGCCCC TTT	GTGTTTCATTTGACGCGGAGTAA	AY19	0.0	0.0
protection	ermT	GTTCACTAGCACTATTTTTAATGACAGAAGT	GAAGGGTGTCTTTTTAATACAATTAACGA	AY137	38.0	2.2
protection	ermT	GTAAAATCCCTAGAGAATACTTTCATCCA	TGAGTGATATTTTTGAAGGGTGTCTT	AY238	36.0	2.2
protection	ermX	GCTCAGTGGTCCCCATGGT	ATCCCCCCGTCAACGTTT	AY209	86.0	7.6
protection	ermY	TTGTCTTTGAAAGTGAAGCAACAGT	TAACGCTAGAGAACGATTTGTATTGAG	AY270	0.0	0.0
efflux	lmrA	TTCAGATGCAATGGCGTTTG	ATAATCGGGAACATAATGAGCATAACTAC	AY77	58.0	0.0
efflux	lmrA	TCGACGTGACCGTAGTGAACA	CGTGACTACCCAGGTGAGTTGA	AY252	0.0	0.0
deactivate	lnuA	TGACGCTCAACACACTCAAAAA	TTCATGCTTAAGTTCCATACGTGAA	AY251	24.0	0.0
deactivate	lnuB	TGAACATAATCCCCTCGTTTAAAGAT	TAATTGCCCTGTTTCATCGTAAATAA	AY67	96.0	62.0
deactivate	lnuB	AAAGGAGAAGGTGACCAATACTCTGA	GGAGCTACGTCAAACAACCAGTT	AY68	96.0	67.4
deactivate	lnuC	TGGTCAATATAACAGATGTAAACCAGATTT	CACCCCAGCCACCATCAA	AY278	0.0	3.3
efflux	matA/mel	TAGTAGGCAAGCTCGGTGTTGA	CCTGTGCTATTTTAAGCCTTGTTTCT	AY139	100.0	82.6
efflux	mefA	CCGTAGCATTGGAACAGCTTTT	AAACGGAGTATAAGAGTGCTGCAA	AY146	96.0	83.7
deactivate	mphA	CTGACGCGCTCCGTGTT	GGTGGTGCATGGCGATCT	AY140	66.0	22.8
deactivate	mphA	TGATGACCCTGCCATCGA	TTCGCGAGCCCCTCTTC	AY142	70.0	28.3
deactivate	mphB	CGCAGCGCTTGATCTTGTAG	TTACTGCATCCATACGCTGCTT	AY141	14.0	0.0
deactivate	mphC	CGTTTGAAGTACCGAATTGGAAA	GCTGCGGGTTTGCCTGTA	AY273	0.0	0.0
efflux	msrA	CTGCTAACACAAGTACGATTCCAAAT	TCAAGTAAAGTTGTCTTACCTACACCATT	AY149	0.0	0.0
efflux	msrA	AACGAAATCAAGCGCAACAA	CAACCGTGCCTTTTTCTTTTG	AY276	0.0	3.3
efflux	msrC	TCAGACCGGATCGGTTGTC	CCTATTTTTTGGAGTCTTCTCTCTAATGTT	AY138	22.0	0.0

efflux	msrC	GAATCACTTGTCCGCAGTTTGTT	CGTACACAACGGTTTCGTCAGA	AY150	0.0	0.0
efflux	oleC	CCCGGAGTCGATGTTCGA	GCCGAAGACGTACACGAACAG	AY285	0.0	3.3
protection	pikR1	TCGACATGCGTGACGAGATT	CCGCGAATTAGGCCAGAA	AY289	0.0	0.0
protection	pikR2	TCGTGGGCCAGGTGAAGA	TTCCCCTTGCCGGTGAA	AY290	0.0	0.0
protection	pncA	GCAATCGAGGCGGTGTTC	TTGCCGCAGCCAATTCA	AY229	2.0	5.4
deactivate	vatB	GGAAAAAGCAACTCCATCTCTTGA	TCCTGGCATAACAGTAACATTCTGA	AY224	4.0	0.0
deactivate	vatB	TTGGGAAAAAGCAACTCCATCT	CAATCCACACATCATTTCCAACA	AY266	4.0	0.0
deactivate	vatC	CGATGTTTGGATTGGACGAGAT	GCTGCAATAATAGCCCCGTTT	AY267	0.0	0.0
deactivate	vatC	CGGAAATTGGGAACGATGTT	GCAATAATAGCCCCGTTTCCTA	AY225	0.0	0.0
deactivate	vatD	TGCAATAGTAGCTGCTAATTCTGTTGTT	TGTTTTATTTCGTTAGCAGGATTTCC	AY73	0.0	0.0
deactivate	vatE	GGTGCCATTATCGGAGCAAAT	TTGGATTGCCACCGACAAT	AY74	0.0	0.0
deactivate	vatE	GACCGTCCTACCAGGCGTAA	TTGGATTGCCACCGACAATT	AY263	0.0	0.0
deactivate	vgb	AGGGAGGGTATCCATGCAGAT	ACCAAATGCGCCCGTTT	AY144	0.0	0.0
deactivate	vgb	CCACGATGGCTGCCTTTG	GGCCATGCAGGACGGATAT	AY145	0.0	0.0
efflux	vgaA	CGAGTATTGTGGAAAGCAGCTAGTT	CCCGTACCGTTAGAGCCGATA	AY226	0.0	0.0
efflux	vgaA	GACGGGTATTGTGGAAAGCAA	TTTCCTGTACCATTAGATCCGATAATT	AY265	0.0	0.0
efflux	vgaB	TAAAAGAGAATAAGGCGCAAGGA	TGTTTAGTAGCATGTTGCATTTTTCC	AY227	4.0	0.0
efflux	vgaB	GAATGATTAAGCCCCCTTCAAAA	ATTCGTGTTTCCAACGATTTCG	AY264	2.0	0.0
deactivate	vgbA	ATACGAGCTGCCTAATAAAGGATCTT	TGTGAACCACAGGGCATTATCA	AY269	0.0	0.0
deactivate	vgbB	CAGCCGGATTCTGGTCCTT	TACGATCTCCATTCAATTGGGTAAA	AY268	0.0	0.0
**Sulphonamides (SUL)**						
protection	folA	CGAGCAGTTCCTGCCAAAG	CCCAGTCATCCGGTTCATAATC	AY208	4.0	0.0
protection	folP	CAGGCTCGTAAATTGATAGCAGAAG	CTTTCCTTGCGAATCGCTTT	AY280	10.0	3.3
protection	folP	GCGATTCGCAAGGAAAGTGA	CACATGGGCCATTTTTTCATC	AY281	10.0	0.0
protection	folP	CACGGCTTCGGCTCATGT	TGCCATCCTGTGACTAGCTACGT	AY282	6.0	0.0
protection	sul1[a]	CGCACCGGAAACATCGCTGCAC	TGAAGTTCCGCCGCAAGGCTCG	AY110	90.0	70.7
protection	sul1	CAGCGCTATGCGCTCAAG	ATCCCGCTGCGCTGAGT	AY178	0.0	0.0
protection	sul1	GCCGATGAGATCAGACGTATTG	CGCATAGCGCTGGGTTTC	AY363	100.0	100.0
protection	sul2	TCATCTGCCAAACTCGTCGTTA	GTCAAAGAACGCCGCAATGT	AY133	96.0	95.7
protection	sul2	TCCGATGGAGGCCGGTATCTGG	CGGGAATGCCATCTGCCTTGAG	AY332	94.0	89.1

protection	sul3	TCCGTTCAGCGAATTGGTGCAG	TTCGTTCACGCCTTACACCAGC	AY333	28.0	3.3
**Tetracycline (TET)**						
protection	tet(32)	CCATTACTTCGGACAACGGTAGA	CAATCTCTGTGAGGGCATTTAACA	AY54	96.0	69.6
deactivate	tet34	CTTAGCGCAAACAGCAATCAGT	CGGTGATACAGCGCGTAAACT	AY321	0.0	0.0
efflux	tet35	ACCCCATGACGTACCTGTAGAGA	CAACCCACACTGGCTACCAGTT	AY320	0.0	0.0
protection	tet(36)	AGAATACTCAGCAGAGGTCAGTTCCT	TGGTAGGTCGATAACCCGAAAAT	AY22	46.0	2.2
protection	tet36	TGCAGGAAAGACCTCCATTACAG	CTTTGTCCACACTTCCACGTACTATG	AY301	68.0	4.3
deactivate	tet37	GAGAACGTTGAAAAGGTGGTGAA	AACCAAGCCTGGATCAGTCTCA	AY302	0.0	0.0
efflux	tet38	TTAATGTGGCGGTATCTGTAGGTATT	TTGCCTGGGAAATTTAATGCTTT	AY271	0.0	0.0
efflux	tetA	GCTGTTTGTTCTGCCGGAAA	GGTTAAGTTCCTTGAACGCAAACT	AY13	0.0	0.0
efflux	tetA	CTCACCAGCCTGACCTCGAT	CACGTTGTTATAGAAGCCGCATAG	AY180	98.0	62.0
efflux	tetA/B	AGTGCGCTTTGGATGCTGTA	AGCCCCAGTAGCTCCTGTGA	AY181	90.0	5.4
efflux	tetA/B	GCCCAGTGCTGTTGTTGTCAT	TGAAAGCAAACGGCCTAAATACA	AY190	94.0	8.7
efflux	tetA(P)	AGTTGCAGATGTGTATAGTCGTAAACTATCTATT	TGCTACAAGTACGAAAACAAAACTAGAA	AY55	62.0	23.9
efflux	tetC	CATATCGCAATACATGCGAAAAA	AAAGCCGCGGTAAATAGCAA	AY182	90.0	4.3
efflux	tetC	ACTGGTAAGGTAAACGCCATTGTC	ATGCATAAACCAGCCATTGAGTAAG	AY199	92.0	3.3
efflux	tetC	TGCGTTGATGCAATTTCTATGC	GGAATGGTGCATGCAAGGAG	AY330	98.0	81.5
efflux	tetD	TGCCGCGTTTGATTACACA	CACCAGTGATCCCGGAGATAA	AY92	20.0	0.0
efflux	tetD	TGTCATCGCGCTGGTGATT	CATCCGCTTCCGGGAGAT	AY188	4.0	0.0
efflux	tetD	CTGGACGCGATGGGAATT	TCCGCTTCCGGGAGATATT	AY349	24.0	0.0
efflux	tetE	TTGGCGCTGTATGCAATGAT	CGACGACCTATGCGATCTGA	AY291	96.0	67.4
efflux	tetG	TCAACCATTGCCGATTCGA	TGGCCCGGCAATCATG	AY183	88.0	64.1
efflux	tetG	CATCAGCGCCGGTCTTATG	CCCCATGTAGCCGAACCA	AY189	92.0	89.1
efflux	tetH	TTTGGGTCATCTTACCAGCATTAA	TTGCGCATTATCATCGACAGA	AY187	64.0	2.2
efflux	tetJ	GGGTGCCGCATTAGATTACCT	TCGTCCAATGTAGAGCATCCATA	AY240	0.0	0.0
efflux	tetK	CAGCAGTCATTGGAAAATTATCTGATTATA	CCTTGTACTAACCTACCAAAAATCAAAATA	AY184	0.0	0.0
efflux	tetL	AGCCCGATTTATTCAAGGAATTG	CAAATGCTTTCCCCCTGTTCT	AY194	0.0	0.0
efflux	tetL	ATGGTTGTAGTTGCGCGCTATAT	ATCGCTGGACCGACTCCTT	AY195	54.0	3.3
protection	tetM	CATCATAGACACGCCAGGACATAT	CGCCATCTTTTGCAGAAATCA	AY186	100.0	78.3
protection	tetM	TAATATTGGAGTTTTAGCTCATGTTGATG	CCTCTCTGACGTTCTAAAAGCGTATTAT	AY193	96.0	65.2

protection	tetM	GCAATTCTACTGATTTCTGC	CTGTTTGATTACAATTTCCGC	AY329	98.0	43.5
protection	tetM[a]	GCAATTCTACTGATTTCTGC	CTGTTTGATTACAATTTCCGC	AY357	58.0	31.5
protection	tetO	ATGTGGATACTACAACGCATGAGATT	TGCCTCCACATGATATTTTTCCT	AY179	98.0	83.7
protection	tetO	CAACATTAACGGAAAGTTTATTGTATACCA	TTGACGCTCCAAATTCATTGTATC	AY192	98.0	79.3
protection	tetPB	ACACCTGGACACGCTGATTTT	ACCGTCTAGAACGCGGAATG	AY292	0.0	1.1
protection	tetPB	TGATACACCTGGACACGCTGAT	CGTCCAAAACGCGGAATG	AY293	0.0	0.0
protection	tetPB	TGGGCGACAGTAGGCTTAGAA	TGACCCTACTGAAACATTAGAAATATACCT	AY294	46.0	19.6
protection	tetPB	AGTGGTGCAAATACTGAAAAAGTTGT	TTTGTTCCTTCGTTTTGGACAGA	AY295	0.0	0.0
protection	tetPB	CTGAAGTGGAGCGATCATTCC	CCCTCAACGGCAGAAATAACTAA	AY296	0.0	0.0
protection	tetQ	CGCCTCAGAAGTAAGTTCATACACTAAG	TCGTTCATGCGGATATTATCAGAAT	AY185	100.0	89.1
regulator	tetR	ATGAGTTCGGCCAGAATTTCC	GGTTGTGCGCGAAATGATT	AY135	0.0	0.0
regulator	tetR	CGCGATAGACGCCTTCGA	TCCTGACAACGAGCCTCCTT	AY197	98.0	50.0
regulator	tetR	CGCGATGGAGCAAAAGTACAT	AGTGAAAAACCTTGTTGGCATAAAA	AY198	94.0	5.4
regulator	tetR	CAATCCATCGACAATCAC	GACAATCAGCTACTTCAC	AY381	90.0	37.0
protection	tetS	TTAAGGACAAACTTTCTGACGACATC	TGTCTCCCATTGTTCTGGTTCA	AY200	58.0	0.0
protection	tetT	CCATATAGAGGTTCCACCAAATCC	TGACCCTATTGGTAGTGGTTCTATTG	AY297	42.0	3.3
unknown	tetU	GTGGCAAAGCAACGGATTG	TGCGGGCTTGCAAAACTATC	AY69	6.0	0.0
unknown	tetU	AACAGCGGGTTAAGTGTGCAA	ATGGTATCATTCAGTTTTCCGACAAT	AY70	0.0	0.0
efflux	tetV	GCGGGAACGACGATGTATATC	CCGCTATCTCACGACCATGAT	AY228	0.0	0.0
protection	tetW	ATGAACATTCCCACCGTTATCTTT	ATATCGGCGGAGAGCTTATCC	AY191	98.0	91.3
deactivate	tetX	AAATTTGTTACCGACACGGAAGTT	CATAGCTGAAAAAATCCAGGACAGTT	AY196	98.0	88.0
**Vancomycin (VAN)**						
protection	vanA	AAAAGGCTCTGAAAACGCAGTTAT	CGGCCGTTATCTTGTAAAAACAT	AY210	2.0	0.0
protection	vanA[a]	TCTGCAATAGAGATAGCCGC	GGAGTAGCTATCCCAGCATT	AY368	2.0	0.0
protection	vanA3FP[a]	CTGTGAGGTCGGTTGTGCG	TTTGGTCCACCTCGCCA	AY4	12.0	27.2
protection	vanB	TTGTCGGCGAAGTGGATCA	AGCCTTTTTCCGGCTCGTT	AY211	0.0	0.0
protection	vanB	CCGGTCGAGGAACGAAATC	TCCTCCTGCAAAAAAAGATCAAC	AY212	8.0	0.0
protection	vanB	GGCTGTTTCGGGCTGTGA	AACAACTAACGCGGCACTGTT	AY354	2.0	0.0
protection	vanC	ACAGGGATTGGCTATGAACCAT	TGACTGGCGATGATTTGACTATG	AY66	10.0	0.0
protection	vanC	CCTGCCACAATCGATCGTT	CGGCTTCATTCGGCTTGATA	AY71	0.0	0.0

protection	vanC	AAATCAATACTATGCCGGGCTTT	CCGACCGCTGCCATCA	AY72	48.0	34.8
protection	vanC	AGGCGATAGCGGGTATTGAA	CAATCGTCAATTGCTCATTTCC	AY303	0.0	0.0
protection	vanC2/vanC3	TTTGACTGTCGGTGCTTGTGA	TCAATCGTTTCAGGCAATGG	AY304	28.0	0.0
protection	vanD	CAGAGGAACATAATGTTTCGATAAAATCT	GCCGGATTTTGTGATTCCAA	AY213	10.0	0.0
protection	vanG	ATTTGAATTGGCAGGTATACAGGTTA	TGATTTGTCTTTGTCCATACATAATGC	AY305	0.0	0.0
protection	vanHB	GAGGTTTCCGAGGCGACAA	CTCTCGGCGGCAGTCGTAT	AY215	4.0	3.3
protection	vanHD	GTGGCCGATTATACCGTCATG	CGCAGGTCATTCAGGCAAT	AY214	6.0	0.0
protection	vanRA	CCCTTACTCCCACCGAGTTTT	TTCGTCGCCCCATATCTCAT	AY216	2.0	0.0
protection	vanRA	CCACTCCGGCCTTGTCATT	GCTAACCACATTCCCCTTGTTTT	AY217	2.0	0.0
protection	vanRB	GCCCTGTCGGATGACGAA	TTACATAGTCGTCTGCCTCTGCAT	AY306	0.0	0.0
protection	vanRC	TGCGGGAAAAACTGAACGA	CCCCCCATACGGTTTTGATTA	AY307	0.0	0.0
protection	vanRC4	AGTGCTTTGGCTTATCTCGAAAA	TCCGGCAGCATCACATCTAA	AY308	20.0	0.0
protection	vanRD	TTATAATGGCAAGGATGCACTAAAGT	CGTCTACATCCGGAAGCATGA	AY309	4.0	0.0
protection	vanSA	CGCGTCATGCTTTCAAAATTC	TCCGCAGAAAGCTCAATTTGTT	AY218	2.0	0.0
protection	vanSB	GCGCGGCAAATGACAAC	TTTGCCATTTTATTCGCACTGT	AY310	8.0	3.3
protection	vanSC	ATCAACTGCGGGAGAAAAGTCT	TCCGCTGTTCCGCTTCTT	AY311	0.0	0.0
protection	vanSC	GCCATCAGCGAGTCTGATGA	CAGCTGGGATCGTTTTTCCTT	AY312	2.0	0.0
protection	vanSE	TGGCCGAAGAAGCAGGAA	CAATAATACTCGTCAAAGGAGTTCTCA	AY313	0.0	0.0
protection	vanTC	CACACGCATTTTTTCCCATCTAG	CAGCCAACAGATCATCAAAACAA	AY161	18.0	0.0
protection	vanTC	GTGGTGCCAAGGAAGTTGCT	CGTAGCCACCGCAAAAAAAT	AY314	0.0	0.0
protection	vanTC	ACAGTTGCCGCTGGTGAAG	CGTGGCTGGTCGATCAAAA	AY315	4.0	0.0
protection	vanTG	CGTGTAGCCGTTCCGTTCTT	CGGCATTACAGGTATATCTGGAAA	AY316	40.0	0.0
protection	vanWB	CGGACAAAGATACCCCCTATAAAG	AAATAGTAAATTGCTCATCTGGCACAT	AY220	8.0	0.0
protection	vanWG	ACATTTTCATTTTGGCAGCTTGTAC	CCGCCATAAGAGCCTACAATCT	AY219	54.0	0.0
protection	vanXA	CGCTAAATATGCCACTTGGGATA	TCAAAAGCGATTCAGCCAACT	AY221	2.0	0.0
protection	vanXB	AGGCACAAAATCGAAGATGCTT	GGGTATGGCTCATCAATCAACTT	AY223	16.0	0.0
protection	vanXD	TAAACCGTGTTATGGGAACGAA	GCGATAGCCGTCCCATAAGA	AY222	4.0	0.0
protection	vanYB	GGCTAAAGCGGAAGCAGAAA	GATATCCACAGCAAGACCAAGCT	AY317	4.0	0.0
protection	vanYD	AAGGCGATACCCTGACTGTCA	ATTGCCGGACGGAAGCA	AY318	10.0	0.0
protection	vanYD	CAAACGGAAGAGAGGTCACTTACA	CGGACGGTAATAGGGACTGTTC	AY319	8.0	0.0

**Other**						
protection	mcr-1[a]	ACACTTATGGCACGGTCTATG	GCACACCCAAACCAATGATAC	AY80	0.0	0.0
deactivate	dfrA1	GGAATGGCCCTGATATTCCA	AGTCTTGCGTCCAACCAACAG	AY58	96.0	48.9
deactivate	dfrA1	TTCAGGTGGTGGGGAGATATAC	TTAGAGGCGAAGTCTTGGGTAA	AY334	0.0	0.0
deactivate	dfrA12	CCTCTACCGAACCGTCACACA	GCGACAGCGTTGAAACAACTAC	AY59	86.0	12.0
deactivate	merA	GTGCCGTCCAAGATCATG	GGTGGAAGTCCAGTAGGGTGA	AY331	82.0	82.6
deactivate	bacA	CGGCTTCGTGACCTCGTT	ACAATGCGATACCAGGCAAAT	AY157	10.0	1.1
deactivate	bacA	TTCCACGACACGATTAAGTCATTG	CGGCTCTTTCGGCTTCAG	AY158	96.0	34.8
protection	fabK	TTTCAGCTCAGCACTTTGGTCAT	AAGGCATCTTTTTCAGCCAGTTC	AY279	0.0	3.3
deactivate	fosB	TCACTGTAACTAATGAAGCATTAGACCAT	CCATCTGGATCTGTAAAGTAAAGAGATC	AY143	0.0	0.0
deactivate	fosX	GATTAAGCCATATCACTTTAATTGTGAAAG	TCTCCTTCCATAATGCAAATCCA	AY76	0.0	0.0
regulator	imiR	CCGGACTAGAGCTTCATGTAAGC	CCCACGCGGTACTCTTGTAAA	AY65	0.0	0.0
deactivate	nimE	TGCGCCAAGATAGGGCATA	GTCGTGAATTCGGCAGGTTTA	AY152	50.0	0.0
deactivate	nisB	GGGAGAGTTGCCGATGTTGTA	AGCCACTCGTTAAAGGGCAAT	AY79	6.0	0.0
deactivate	nisB	CGGTTGAACGGCGTGAA	TTTCCACCCAGGTTTGCTACTATT	AY348	14.0	0.0
deactivate	yyaR	CCGTTGCAAGAAGATTATAGAAAAAA	CAAGCATAAGACCGCATAAATGAT	AY16	0.0	0.0
**Integrase**						
MGE	intI1[a]	GATCGGTCGAATGCGTGT	GCCTTGATGTTACCCGAGAG	AY45	100.0	97.8
MGE	intI1	CGAAGTCGAGGCATTTCTGTC	GCCTTCCAGAAAACCGAGGA	AY336	100.0	97.8
MGE	intI1	GCCTTGATGTTACCCGAGAG	GATCGGTCGAATGCGTGT	AY337	96.0	95.7
MGE	intI1	CGAACGAGTGGCGGAGGGTG	TACCCGAGAGCTTGGCACCCA	AY359	96.0	94.6
MGE	intI1	CTGGATTTCGATCACGGCACG	ACATGCGTGTAAATCATCGTCG	AY364	86.0	84.8
MGE	intI2	TGCTTTTCCCACCCTTACC	GACGGCTACCCTCTGTTATCTC	AY338	96.0	15.2
MGE	intI3	GCCACCACTTGTTTGAGGA	GGATGTCTGTGCCTGCTTG	AY339	94.0	66.3
**Transposase**						
MGE	IS613	AGGTTCGGACTCAATGCAACA	TTCAGCACATACCGCCTTGAT	AY26	68.0	75.0
MGE	tnpA	GCCGCACTGTCGATTTTTATC	GCGGGATCTGCCACTTCTT	AY201	100.0	92.4
MGE	tnpA	CCGATCACGGAAAGCTCAAG	GGCTCGCATGACTTCGAATC	AY202	98.0	95.7
MGE	tnpA	GGGCGGGTCGATTGAAA	GTGGGCGGGATCTGCTT	AY203	92.0	90.2
MGE	tnpA	CATCATCGGACGGACAGAATT	GTCGGAGATGTGGGTGTAGAAAGT	AY204	90.0	78.3

MGE	tnpA	GAAACCGATGCTACAATATCCAATTT	CAGCACCGTTTGCAGTGTAAG	AY205	100.0	91.3
MGE	tnpA	TGCAGATGGTTTAACCTTGGATATTT	TCGGTTCATCAAACTGCTTCAC	AY206	96.0	66.3
MGE	tnpA	AATTGATGCGGACGGCTTAA	TCACCAAACTGTTTATGGAGTCGTT	AY207	96.0	81.5
MGE	Tp614	GGAAATCAACGGCATCCAGTT	CATCCATGCGCTTTTGTCTCT	AY25	100.0	80.4
MGE	trfA	ACGAAGAAATGGTTGTCCTGTTC	CGTCAGCTTGCGGTACTTCTC	AY358	78.0	66.3
**Insertional sequence**						
MGE	IS1111	GTCTTAAGGTGGGCTGCGTG	CCCCGAATCTCATTGATCAGC	AY376	0.0	1.1
MGE	IS1133	GCAGCGTCGGGTTGGA	ACGCGTTCGAACAACTGTAATG	AY383	82.0	2.2
MGE	ISAba3	TCAGAGGCAGCGGTATACGA	GGTTGATTCAGTTAAAGTACGTAAAACTTT	AY371	100.0	91.3
MGE	ISEfm1	AGGTGTCCATGACGTGAAAGTG	TCCTTTGTCCCCTAGGATATTGG	AY372	92.0	5.4
MGE	ISPps	CACACTGCAAAAACGCATCCT	TGTCTTTGGCGTCACAGTTCTC	AY369	100.0	95.7
MGE	ISSm2	TGGATCGACCGGTTCCAT	GCTGACCGAGCTGTCCATGT	AY370	100.0	100.0
MGE	orf37-IS26	GCCGGGTTGTGCAAATAGAC	TGGCAATCTGTCGCTGCTG	AY365	50.0	0.0
MGE	orf39-IS26	GCGCGTCGAGCATCAATAG	CAGTTGTGCTGCTGGTGGTC	AY366	82.0	5.4
MGE	Tn5	CAGCATAAAAAATCCCGACAACA	CCCCGCAACAGACATACGT	AY384	48.0	0.0
**Plasmid-associated**						
MGE	IncN_rep	AGTTCACCACCTACTCGCTCCG	CAAGTTCTTCTGTTGGGATTCCG	AY340	n.a.	n.a.
MGE	IncN_oriT	TTGGGCTTCATAGTACCC	GTGTGATAGCGTGATTTATGC	AY341	n.a.	n.a.
MGE	IncP_oriT	CAGCCTCGCAGAGCAGGAT	CAGCCGGGCAGGATAGGTGAAGT	AY342	n.a.	n.a.
MGE	IncQ_oriT	TTCGCGCTCGTTGTTCTTCGAGC	GCCGTTAGGCCAGTTTCTCG	AY343	n.a.	n.a.
MGE	IncW_trwAB	AGCGTATGAAGCCCGTGAAGGG	AAAGATAAGCGGCAGGACAATAACG	AY344	n.a.	n.a.
MGE	PAMBL	CAGGCTCTTAATGTGATA	TTATGCTCAATACTCGTG	AY377	n.a.	n.a.
MGE	pAKD1	GGTAAGATTACCGATAAACT	GTTCGTGAAGAAGATGTA	AY380	n.a.	n.a.
MGE	pNI105	CGCTAAGGATGTTTACAC	CTCAACCGTTCTAGGATT	AY379	n.a.	n.a.
MGE	repA	CCCCCAGGACTTGCGAGCG	GAGGCATGCACGCCGACCA	AY378	n.a.	n.a.

n.a. not available

Note: Plasmid-associated genes, due to their low representativeness, were excluded from the analyses.

Table S3. Consumption of antibacterials for systemic use (ATC group J01) in the community (primary care sector) in different European countries from 2005 to 2015 (see also Fig. 1), defined as daily dose per 1000 inhabitants and per day.

Year	Portugal (PT)	Spain (ES)	Cyprus* (CYP)	Ireland (IL)	Germany (DE)	Finland (FI)	Norway (NO)
2005	24.5	19.3	-	20.5	14.6	18.1	16.8
2006	22.7	18.7	31.9	21.2	13.6	17.4	14.8
2007	–	19.9	33.9	22.9	14.5	18.3	15.5
2008	22.7	19.8	33.7	22.5	14.6	17.8	15.6
2009	22.9	19.7	34.4	20.8	14.9	17.9	15.2
2010	22.4	20.3	31.0	20.3	14.5	18.5	15.8
2011	23.2	20.9	32.0	22.6	14.1	20.1	16.5
2012	22.7	19.7	29.7	23.0	14.9	19.5	16.9
2013	19.6	20.3	28.2	23.8	15.8	18.3	16.2
2014	20.3	21.6	26.1	23.1	14.6	18.1	15.9
2015	21.3	22.2	31.1	25.6	14.4	17.2	15.8
Mean value 2005-2015	22.2	20.2	31.2	22.4	14.6	18.3	15.9

* includes total care cases only

Source: https://ecdc.europa.eu/en/antimicrobial-consumption/database/trend-country

Table S4. Characterization of the UWTPs examined in this study, in terms of dimension, geographic conditions, treatment, and microbiological indicators.

UWTP	Treatment (sT or tT)	Avg. air annual temp. (ºC)	Wastewater temp. (ºC) Spring (S) Autumn (A)	Population equivalent	N.º of hospitals/hospital beds in the area	BOD5 (Effluent)	COD (Effluent)	HRT (hours)	SRT (days)	DWF (m^3/day)
**PT1***	sT	15	S: n.a.<br>A: 21	160 000	n.a./n.a.	15−25	78−107	11	2.8-5.3	21 241-21 884
**PT2**	sT	15	S: 19<br>A: 20-24	150 094	1/1120	5.0−11	34−50	n.a.	n.a.	32 060-34 005
**PT3**	sT + UV disinfection	15	S: 18	173 839	n.a./n.a.	8.0	29	n.a.	12	n.a.-23 793
**ES1**	sT	15	S: 18<br>A: 24	200 000	3/400	1.4−7.9	n.a.	24	18	43 000
**CYP1**	tT	20	S: 22<br>A: 26-30	151 000	n.a./n.a.	4.0−12	23−33	25	8.9	18 000-21 480
**CYP2**	tT	20	S: 21-23<br>A: 28-31	30 000	n.a./n.a.	2.0−26	18−42	18	30	4 000
**IL1**	tT	10	n.a.	2 000 – 100 000	n.a.	n.a.	n.a.	n.a.	n.a.	1 000 – 30 000
**IL2**	tT	10	n.a.	2 000 – 100 000	n.a.	n.a.	n.a.	n.a.	n.a.	1 000 – 30 000
**DE1**	sT	9	S: 11-12<br>A: 17-19	440 000	3/2162	2.0	12−17	12	12	114 221-126 317
**DE2**	sT	11	n.a.	650 000	8/5048	<3.0−5.0	20−34	29	14	137 000-140 600
**FI1**	tT	6	S: 12<br>A: 17	800 000	11/2492	3.9−4.4	41−52	20	14	270 684-278 082
**FI2**	sT	6	S: 10<br>A: 16	310 000	2/812	3.7−5.4	26−40	19	13	100 273-102 191
**NO1**	sT	7	S: 8<br>A: 15-16	739 000	4/2650	6.0−18	41−54	3.5	25-37	250 959-272 877

sT, secondary treatment; tT, tertiary treatment;
Avg. temp., annual average temperature for the region according to the timeanddate.com (www. timeanddate.com).
HRT, hydraulic residence time; SRT, sludge residence time; DWF, daily water flow; n.a. – not available.
*Soon after this study, PT1 was temporarily closed for improvement restoration.

Table S4. (continuation)

UWTP	Faecal coliforms counts (log CFU's/mL) in final effluent				Enterococci counts (log CFU's/mL) in final effluent			
		**AML_R**	**TET_R**	**CIP_R**		**AML_R**	**TET_R**	**CIP_R**
**PT1**	6.22±0.56[a]	5.91±0.43[a]	4.95±0.46[a]	5.12±0.40[a]	5.01±0.56[a]	2.87±0.50[a]	4.32±0.58[a]	3.41±0.38[a]
**PT2**	5.13±0.44[ab]	4.81±0.51[ab]	3.93±0.35[ab]	4.33±0.51[ab]	4.17±0.61[ab]	2.06±0.75[abcd]	3.37±0.40[ab]	2.44±0.68[abc]
**PT3**	3.61±0.18[cd]	3.09±0.03[cd]	2.45±0.20[bcd]	2.67±0.11[bcd]	2.91±0.19[bc]	0.86±0.06[bcd]	2.14±0.23[bcd]	1.43±0.06[abcd]
**ES1**	4.87±0.62[bc]	4.36±0.80[bc]	4.10±1.02[ab]	4.12±0.63[abcd]	3.85±0.36[ab]	3.45±0.16[ab]	2.79±0.34[bcdef]	3.06±0.48[abcd]
**CYP1**	3.00±1.71[de]	2.13±0.85[de]	2.11±1.61[cde]	2.45±1.62[cde]	1.77±1.38[cd]	1.29±0.11[cd]	1.08±1.59[def]	1.45±1.38[bcd]
**CYP2**	2.31±0.61[de]	1.61±0.29[ef]	1.08±0.72[de]	1.52±0.68[de]	0.59±0.40[d]	≤ -2.7	-0.30±0.32[f]	0.18±0.44[d]
**IL1**	4.50±0.25[bc]	4.10±0.11[bc]	3.08±0.39[abc]	3.45±0.40[abcd]	3.17±0.26[b]	≤ 0.00	2.21±0.13[bcdef]	≤ 0.00
**IL2**	4.63±0.36[bc]	4.27±0.24[bc]	3.14±0.42[ab]	3.51±0.47[abc]	3.33±0.27[ab]	< 0.00	2.35±0.25[cdef]	≤ 0.00
**DE1**	1.54±0.82[ef]	0.95±0.46[f]	0.52±0.33[e]	0.75±0.62[e]	1.55±0.01[d]	0.30±0.06[cd]	0.75±0.00[ef]	≤ -2.00
**DE2**	5.05±0.31[abc]	4.86±0.24[ab]	3.85±0.33[bc]	3.72±0.49[bcd]	4.91±0.31[ab]	3.63±0.25[ab]	3.86±0.23[abc]	4.30±0.18[ab]
**FI1**	n.a.	n.a.	n.a.	n.a.	n.a.	n.a.	n.a.	n.a.
**FI2**	n.a.	n.a.	n.a.	n.a.	n.a.	n.a.	n.a.	n.a.
**NO1**	≤ 2.00	≤ 1.00	≤ 1.00	≤ 1.00	3.49±0.60[bc]	2.80±0.32[abc]	≤ 0.00	≤ 0.00

a-f: significant differences among wastewater treatment plants, determined based on ANOVA and the post-hoc Tuckey test ($p < 0.01$).
AML_R, amoxicillin resistant; TET_R, tetracycline resistant; CIP_R, ciprofloxacin resistant;
n.a. not available
